# Heat-stress triggers MAPK crosstalk to turn on the hyperosmotic response pathway

**DOI:** 10.1038/s41598-018-33203-6

**Published:** 2018-10-11

**Authors:** Paula Dunayevich, Rodrigo Baltanás, José Antonio Clemente, Alicia Couto, Daiana Sapochnik, Gustavo Vasen, Alejandro Colman-Lerner

**Affiliations:** 1 Departamento de Fisiología, Biología Molecular y Celular, Facultad de Ciencias Exactas y Naturales (FCEN), Universidad de Buenos Aires (UBA), Buenos Aires, Argentina; 2 Instituto de Fisiología, Biología Molecular y Neurociencias (IFIBYNE), CONICET-UBA, Buenos Aires, Argentina; 3 CIHIDECAR-Departamento de Química Orgánica, FCEN, UBA, Buenos Aires, Argentina

## Abstract

Cells make decisions based on a combination of external and internal signals. In yeast, the high osmolarity response (HOG) is a mitogen-activated protein kinase (MAPK) pathway that responds to a variety of stimuli, and it is central to the general stress response. Here we studied the effect of heat-stress (HS) on HOG. Using live-cell reporters and genetics, we show that HS promotes Hog1 phosphorylation and Hog1-dependent gene expression, exclusively via the Sln1 phosphorelay branch, and that the strength of the activation is larger in yeast adapted to high external osmolarity. HS stimulation of HOG is indirect. First, we show that HS causes glycerol loss, necessary for HOG activation. Preventing glycerol efflux by deleting the glyceroporin *FPS1* or its regulators *RGC1* and *ASK10*/*RGC2*, or by increasing external glycerol, greatly reduced HOG activation. Second, we found that HOG stimulation by HS depended on the operation of a second MAPK pathway, the cell-wall integrity (CWI), a well-known mediator of HS, since inactivating Pkc1 or deleting the MAPK *SLT2* greatly reduced HOG activation. Our data suggest that the main role of the CWI in this process is to stimulate glycerol loss. We found that in yeast expressing the constitutively open channel mutant (Fps1-Δ11), HOG activity was independent of Slt2. In summary, we suggest that HS causes a reduction in turgor due to the loss of glycerol and the accompanying water, and that this is what actually stimulates HOG. Thus, taken together, our findings highlight a central role for Fps1, and the metabolism of glycerol, in the communication between the yeast MAPK pathways, essential for survival and reproduction in changing environments.

## Introduction

Sensing and responding to the environment is critical for survival. In eukaryotic cells, mitogen-activated protein kinase (MAPK) pathways mediate the response to many different stimuli. The budding yeast *S*. *cerevisiae* contains five such pathways^1^. Two of them, the High Osmolarity Glycerol (HOG)^2^ and the Cell Wall Integrity (CWI)^3^ pathways are activated by a variety of environmental changes/conditions. The other three, the pheromone response^4^, the filamentation^5^ and the sporulation/meiosis pathways^6^ control cell fate decisions in response to mating pheromone or nutritional conditions.

**Figure 1:**
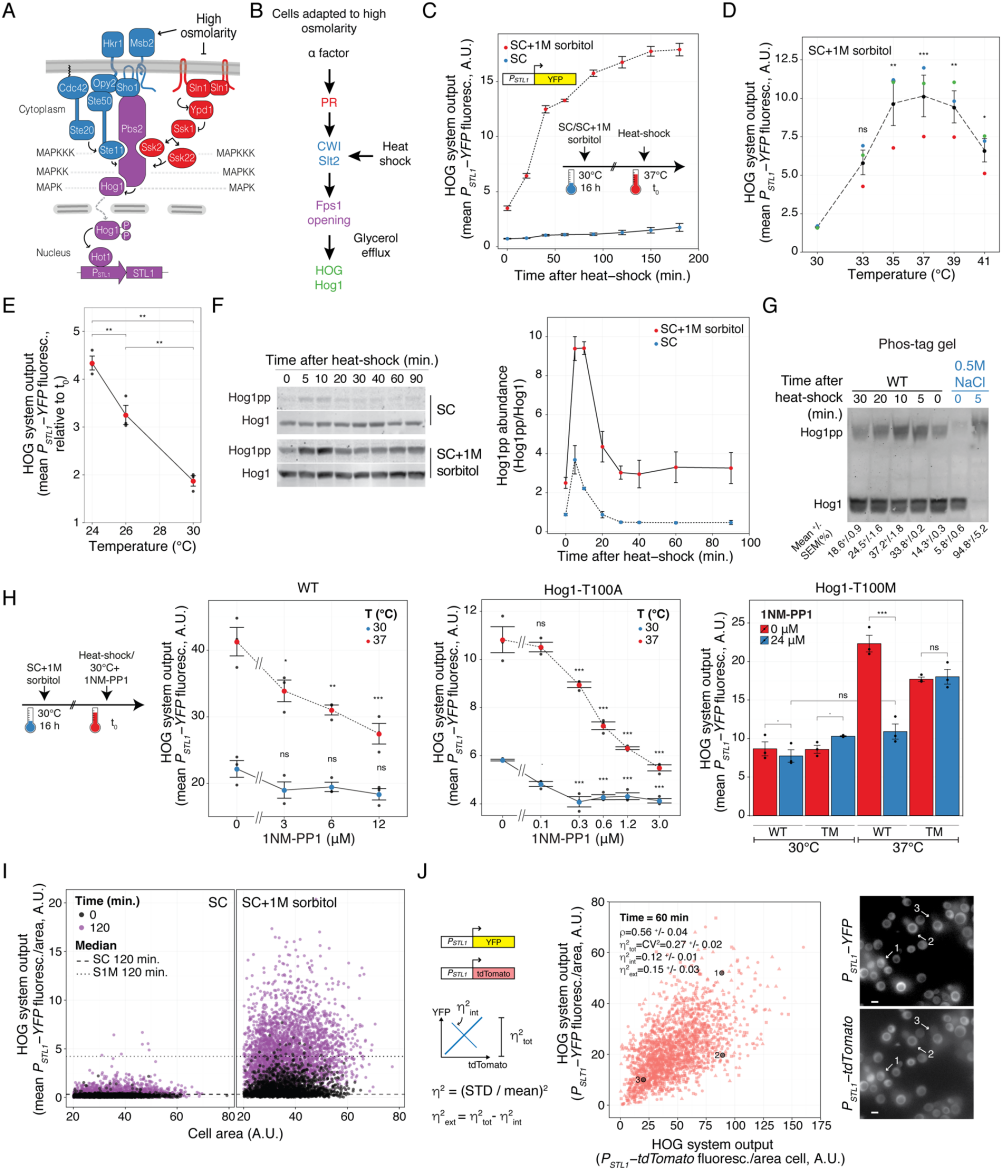
Heat-shock stimulates the HOG pathway. (**A**) The HOG pathway with its two branches (Sho1 in blue and Sln1 in red) converge on Pbs2, which phosphorylates and thus activates the MAPK Hog1. Active Hog1 translocates to the nucleus where it induces gene expression via the transcription factors Sko1, Skp1 (not shown), and Hot1. (**B**) Mating pheromone stimulates HOG indirectly. α factor activates the Cell Wall Integrity pathway, which in turn causes loss of glycerol through the aqua glyceroporin Fps1, leading to reduced turgor, and causing HOG activation. We hypothesize that heat-shock (HS) stimulates HOG via this route as well. This activating pathway is amplified in cells adapted to high osmolarity medium. (**C**) HOG transcriptional reporter dynamics following a shift from 30 °C to 37 °C in cells grown in SC or SC + 1M sorbitol. Plot shows HOG system output (population average of the total YFP accumulated in each cell) vs. time. (**D**) HOG system output of cells grown in SC + 1M sorbitol after 1 h of temperature shift from 30 °C to the indicated temperatures. (**E**) HOG activation by temperature shifts from the indicated temperatures to 37 °C in cells adapted to SC + 1M sorbitol. Plot shows HOG system output after 2 h of temperature shift. Colors correspond to replicate experiments. (**F**) HOG MAPK activation dynamics following a shift from 30 °C to 37 °C. Hog1 phosphorylation was assayed by immunoblotting. Left. Representative blot. Right. Plot of Hog1pp abundance. Uncropped blot in Fig. S3. (**G**) Same as in F but extracts were run in phos-tag polyacrylamide gels. We include a control of an extract from yeast grown in SC 5 minutes after addition of 0.5 M NaCl. Top. Representative blot. Bottom. Quantification showing fraction of phosphorylated Hog1 ± SEM. Uncropped blot in Fig. S4. (**H**) HOG system output in strains expressing WT Hog1, Hog1-T100A or Hog1-T100M mutants was measured after 2 h of temperature shift in the presence of the indicated concentration of 1NM-PP1. Statistical comparisons of left and middle plots are against the 0 μM 1NM-PP1 at the corresponding temperate. (**I**) Scatter plot comparing data at 0 vs. 120 minutes after temperature shift of cells grown in SC or SC + 1 M sorbitol. Plot shows HOG system output/area vs. area of individual cells. (**J**) Cell-to-cell variability in HOG activation. A strain with two identical *STL1* promoters driving YFP or tdTomato grown in 1 M sorbitol medium after 1 h shift from 30 °C to 37 °C. Plot shows YFP vs. tdTomato protein reporters. ρ indicates Pearson correlation coefficient. Total variability is measured using η^2^_tot_ (STD^2^_(xFP/<xFP>)_); intrinsic noise η^2^_int_ (0.5 x STD^2^_(YFP/<YFP> - tdTomato/<tdTomato>)_) and η^2^_ext_ (η^2^_tot_ - η^2^_int_) (see Methods). Images show a representative field. Numbers mark selected cells from the plot. White bar corresponds to 5 μm. Strains: LD3342, RB3937, RB3938 and YGB5938. In panels C, D, E, F, G and H data correspond to the mean of three independent replicates (shown as dots) ± SEM.

HOG responds to many stimuli, including acetic acid^7^, arsenite^8^, cold shock^9^, and heat-shock^10^. The best-characterized stimulus is a high osmolarity shock. An increase in external osmolarity causes loss of turgor pressure and cell volume due to water efflux, which triggers a homeostatic response leading to the accumulation of a compatible osmolyte, which in glucose containing medium is glycerol. The osmotically driven entry of water leads to cell volume recovery. Two signaling branches, known as Sln1 and Sho1 after their sensors, converge to activate the MAPKK Pbs2, which activates the p38-like MAPK Hog1^11^ (Fig. 1A). In the Sln1 branch, this membrane sensor transduces the signal via Ypd1 and Ssk1 by a phosphorelay mechanism to the partly redundant MAPKKKs Ssk2 and Ssk22^12^, which phosphorylate Pbs2. In the other branch, the mucin-like sensors Msb2 and Hrk1 activate Sho1, which recruits a complex to the plasma membrane that includes Cdc42, promoting the activation of Ste20. Ste20 initiates the MAPK cascade by activating Ste11, which phosphorylates Pbs2^13^. Once active, Hog1 stops glycerol efflux by phosphorylating regulators of the Fps1 glycerol channel, Rgc1 and Rgc2/Ask10. When phosphorylated, these proteins detach from the channel, which in turn closes^14,15^. Hog1 also increases metabolic flow towards glycerol by up-regulating Pfk2 (a subunit of phosphofructokinase); inhibiting Ypk1, an inhibitor of Gpd1, responsible for the first step in the reduction of DHAP into glycerol^16^; and blocking Tdh1, 2 and 3^17^, enzymes which divert the flux away from glycerol production. Phosphorylated Hog1 also translocates to the nucleus where it associates with transcription factors, such as Hot1^18^, to induce a large number of genes^19^, including some encoding enzymes and transporters required for glycerol accumulation^20^.

In contrast, a low osmolarity shock results in the aperture of Fps1, which allows glycerol, accompanied by water, to leave the cell^21^. Therefore, the excessive pressure is alleviated. Opening of Fps1 seems to depend on the CWI MAPK, Slt2^2^. The CWI is activated by other stimuli also, including cell-wall binding compounds (like congo red and calcofluor white), heat-shock and cell-wall remodeling events during the mating response^3^. CWI signaling is typically activated by several mucin-like proteins that operate as sensors connecting the plasma membrane and cell wall, and which display varying specificities for different stimuli. These sensors (Wsc1,2,3, Mid2 and Mtl1) recruit the guanine nucleotide exchange factors Rom1 and Rom2, which activate the small G-protein Rho1. Rho1-GTP then activates the kinase Pkc1, which initiates the MAPK cascade by phosphorylating the MAPKKK Bck1. Bck1 stimulates the MAPKKs Mkk1 and Mkk2, which then activate the MAPK Slt2. Slt2, via transcriptional and post-transcriptional actions, causes remodeling and strengthening of the cell wall. Full activation of Slt2 is achieved, besides the activity of this main pathway, by parallel inputs downstream of Rho1, either to Pkc1, Bck1 or directly to Mkk1/2, depending on each particular stimulus^22^. In particular, activation of Slt2 by heat-stress requires the main pathway and extra input to Mkk1,2, possibly from Cbk1 and Bck2^23^.

Thus, HOG and CWI are activated by opposite changes in external osmolarity. Nevertheless, other types of stimuli can activate both pathways^24^. One example is heat-stress, which elicits a complex response that involves a plethora of simultaneous actions by the cell^25^. Among these responses, it activates both HOG^10^ and CWI^26^, although with different dynamics. While Hog1 shows a transient phosphorylation peak at around 5 minutes, Slt2 is slowly phosphorylated reaching a maximum at around 30 minutes.

In addition, we have previously shown that the HOG pathway is activated during the mating response in high osmolarity^27^. The mechanism that leads to this crosstalk involves the activation of the CWI MAPK Slt2 by the cell shape changes associated with the mating projection formation, followed by the aperture of Fps1 (Fig. 1B). The resulting release of internal glycerol and loss of turgor pressure activates the Sln1 branch of HOG, and this leads to increased production of glycerol. This mechanism of crosstalk results in a high glycerol turn-over rate that allows fine and fast tuning of internal pressure within the yeast cell during mating^27^, a pre-condition for fusion^28^.

If the mating response is capable of activating HOG via the CWI pathway, it is likely that other CWI activating inputs would lead to a similar fate. This rationale might expand the role of the HOG pathway to include many inputs previously not thought to be associated with it. Here we show that both heat-shock and the cell-wall disturbing agent congo red can cause HOG activation, which is strongly amplified when yeast are grown in high osmolarity medium. Similar to the crosstalk with the mating pheromone response, it involves the MAPK Slt2, the glycerol channel Fps1 and the Sln1 branch of HOG.

## Results

### Heat-shock activates HOG in yeast adapted to high osmolarity

Our previous studies on the crosstalk between MAPK pathways indicated that activation of the CWI by mating pheromone causes HOG activation^27^, especially noticeable in yeast pre-adapted to high osmolarity medium. Here we asked if CWI activation was sufficient to turn on HOG (Fig. 1B). To test this idea, we stimulated the CWI by mild heat-shock (shifting the culture from 30 °C to 37 °C)^3^. We monitored HOG activation in individual cells using a transcriptional reporter (*P*_*STL1*_*-YFP*) (Fig. 1C). In SC medium, there was no detectable reporter induction. In contrast, in yeast grown in SC supplemented with 1 M sorbitol, there was a fast increase in YFP synthesis. Accumulation of fluorescent protein approached steady-state after two hours, indicating that around this time, the higher reporter expression was compensated by dilution due to cell division. We detected a similar *P*_*STL1*_*-YFP* induction by heat-shock after adapting cells to 0.5 M NaCl (Fig. S1), indicating that it is the high external osmolarity and not sorbitol *per se* what is required for HOG stimulation.

We tested shifts from 30 °C to various temperatures and found maximum expression of the HOG reporter at 37 °C (Fig. 1D). Higher temperatures resulted in a lower induction. This could be because at increasing temperatures, yeast might accumulate trehalose instead of glycerol^29,30^, a process that is independent of HOG. Activation was even stronger (larger fold-change) when cultures were shifted from 24 °C, instead of 30 °C, to 37 °C (Fig. 1E). This is partly because of a lower basal reporter expression at this lower temperature (Fig. S2).

To confirm that the MAPK Hog1 was activated by heat-shock, we examined Hog1 phosphorylation by western blot after the temperature shift (Figs 1F and S3). In SC, as previously reported^10^, there was a small, fast and transient phosphorylation of Hog1, with a peak at around 5 minutes, after which phosphorylated Hog1 dropped, reaching basal levels 30 minutes after heat-shock. In medium supplemented with 1M sorbitol, Hog1 phosphorylation dynamics in response to heat-shock was similar, but amplified: basal phospho-Hog1 was higher, with a higher peak signal. To confirm that Hog1 was doubly phosphorylated, we used phos-tag polyacrylamide gels. The phos-tag resin interacts with phosphate groups in the proteins during electrophoresis, thereby slowing their mobility, and thus providing a way to separate the unphosphorylated from the phosphorylated forms of Hog1 efficiently^31^. Using these gels, a single antibody against Hog1 may be used to detect all its forms, facilitating the quantification of the actual fraction of phosphorylated Hog1. Two results from these gels are worth mentioning (Figs 1G and S4). First, heat-shock caused a mobility shift identical to the hyperosmolarity shock control, confirming that Hog1 was doubly phosphorylated^31^ during HS. Second, whereas with the osmotic shock Hog1 becames fully phosphorylated (that is, all molecules in every cell, as previously reported^31^), less than 40% did so in response to the temperature shift (Figs 1G and S4). This is consistent with heat-shock being a weaker stimulus, resulting in variable activation at the single cell level. Indeed, even though activation by heat-shock involved the whole population, as evidenced by an overall shift over time in the unimodal distribution of accumulated reporter (Fig. S5), there was a rather large cell to cell variability in the response (STD/MEAN = 0.52 ± 0.2, see below).

Even though Hog1 phosphorylation dropped to initial levels 30 minutes post heat-shock (Fig. 1F), expression of the HOG reporter did not diminish (Fig. 1C). This result suggested that at least a small but significant pool of activated Hog1 remains when cells growing in high osmolarity are exposed to heat-shock and that this pool is enough to maintain gene expression. Alternatively, it could mean that reporter expression did not depend on Hog1. Thus, to verify the requirement of Hog1 activity for gene expression, we used the kinase inhibitor 1NM-PP1, an adenine analog. In most kinases, it is necessary to mutate the gatekeeper amino acid in the ATP binding pocket to a smaller residue to allow 1NM-PP1 to fit inside^32^. However, we noted that Hog1 possesses threonine at this location, a relatively small amino acid, which might allow inhibition of the wild-type (WT) form. Indeed, 12 μM inhibitor largely blocked reporter induction in response to a 0.8 M sorbitol hyperosmotic shock (Fig. S6). This same concentration reduced reporter induction in response to heat-shock in yeast adapted to 1 M sorbitol (Fig. 1H, left), and 24 μM almost completely blocked it (Fig. 1H, right). To control that the effect of the inhibitor was specific to Hog1 (out of the 129 yeast kinases, 14 have Thr and 3 have Ala at this location, the rest have bigger residues), we constructed strains expressing Hog1-as2 (Hog1-T100A), which are more sensitive to 1NM-PP1^17^. This mutant is already hypomorph, exhibiting a reduced activation in response to heat-shock in the absence of inhibitor (Fig. S6). Addition of 3 μM of 1NM-PP1 blocked induction (Fig. 1H, middle). As a further control, we made a new mutant with a bigger amino acid as a gatekeeper, Hog1-T100M. This mutant was resistant to the inhibitor both in response to hyperosmotic shock (Fig. S6) and to heat-shock (Fig. 1H, right). Together, these results confirm that *P*_*STL1*_*-YFP* expression in response to heat-shock requires Hog1 activity.

Taken together, these results indicated that heat-shock stimulates the HOG pathway in cells already adapted to high external osmolarity.

### Single-cell analysis reveals large noise during heat-shock activation of HOG

The transient activation of Hog1 in SC did not result in noticeable reporter expression at the population level (Fig. 1C). However, further inspection of individual cells revealed that a fraction of less than 0.5% of SC grown yeast did induce the HOG reporter to levels comparable to those adapted to 1 M sorbitol (note the cells with outlier abundance of YFP in Fig. 1I, left). This is reminiscent of the behavior of yeast exposed to small hyperosmolarity shocks (~0.1 M NaCl), during which many cells fail to induce *STL1*^33^. Pelet *et al*. showed that expression of *STL1* requires a slow stochastic step of chromatin remodeling. When the stimulus is too short, the pathway turns off in a significant fraction of cells (measured by the exit of Hog1 from the nucleus) before the *STL1* locus had time to be remodeled and therefore became susceptible to induction. Due to the randomness in the timing of remodeling, this step introduced noise in the expression of *STL1*^33^. Thus, we suspect that the reason for the lack of induction in SC is that, as during small hyperosmolarity shocks, Hog1 phosphorylation peak is either too short or too low in amplitude.

To assess the contribution of stochastic processes to the induction of *STL1* in response to temperature, we measured gene expression noise (also referred to as intrinsic noise)^33–35^ 60 minutes after the temperature shift by comparing the induction of two identical *STL1* promoters driving the expression of YFP and tdTomato, respectively (Fig. 1J). The degree to which the signal from these promoters is decorrelated in the population is a measure of this noise. We found a mild correlation between YFP and tdTomato (ρ = 0.56 ± 0.04), indicating a large contribution of gene expression noise. Indeed, this noise accounted for 44% of the overall cell-to-cell variability (η^2^_tot_ = 0.27 ± 0.02 vs. η^2^_int_ = 0.12 ± 0.01; η = STD/mean, see Methods). The remaining variability, known as extrinsic noise, corresponds to the correlated variation between the YFP and tdTomato (η^2^_ext_ = 0.15 ± 0.03).

### The Sln1 branch mediates heat-shock activation of HOG

**Figure 2:**
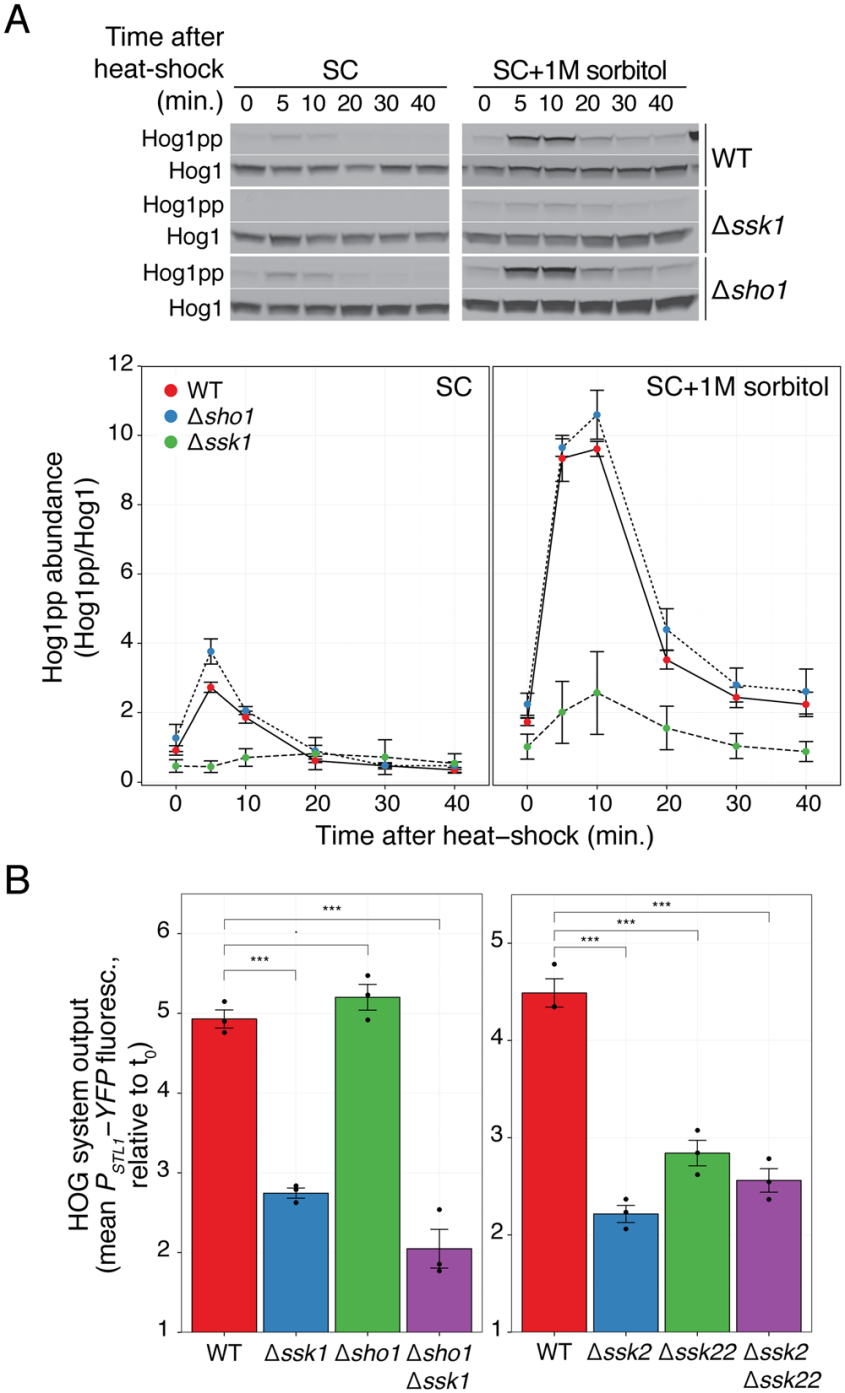
Heat-shock activation of HOG depends on the Sln1 branch. (**A**) HOG MAPK activation dynamics following a shift from 30 °C to 37 °C in branch mutants in SC or in SC + 1 M sorbitol. Top. Representative blot. Uncropped blot in Fig. S7. (**B**) HOG transcriptional reporter in branch mutants measured 2 h after 1 M sorbitol-adapted cells were shifted from 30 °C to 37 °C. Values correspond to the mean of three independent replicates (shown as dots) ± SEM, relative to t_0_. Strains: LD3342, RB3382, RB3704, YPD5937, RB3642, RB3691 and RB3695.

Next, we tested which of the two HOG branches (Fig. 1A) mediates Hog1 activation by heat-shock. Previous reports pointed to the Sho1 branch^10^. However, deletion of *SHO1* did not prevent Hog1 phosphorylation after heat-shock, neither in SC nor in yeast adapted to medium with 1 M sorbitol (Figs 2A and S7). In contrast, deletion of *SSK1* greatly diminished Hog1 phosphorylation, both in SC and in 1 M sorbitol. Consistent with this result, the *P*_*STL1*_*-YFP* reporter was unaffected in *Δsho1* cells but was significantly reduced in *Δssk1*, *Δssk2*, *Δssk22* and *Δssk2Δssk22* cells (Fig. 2B). The double knockout *Δssk1Δsho1* showed an even lower reporter expression, suggesting a minor role for the Sho1 branch when the Sln1 branch is inactivated.

### Heat-shock activation of HOG requires the CWI MAPK Slt2

**Figure 3:**
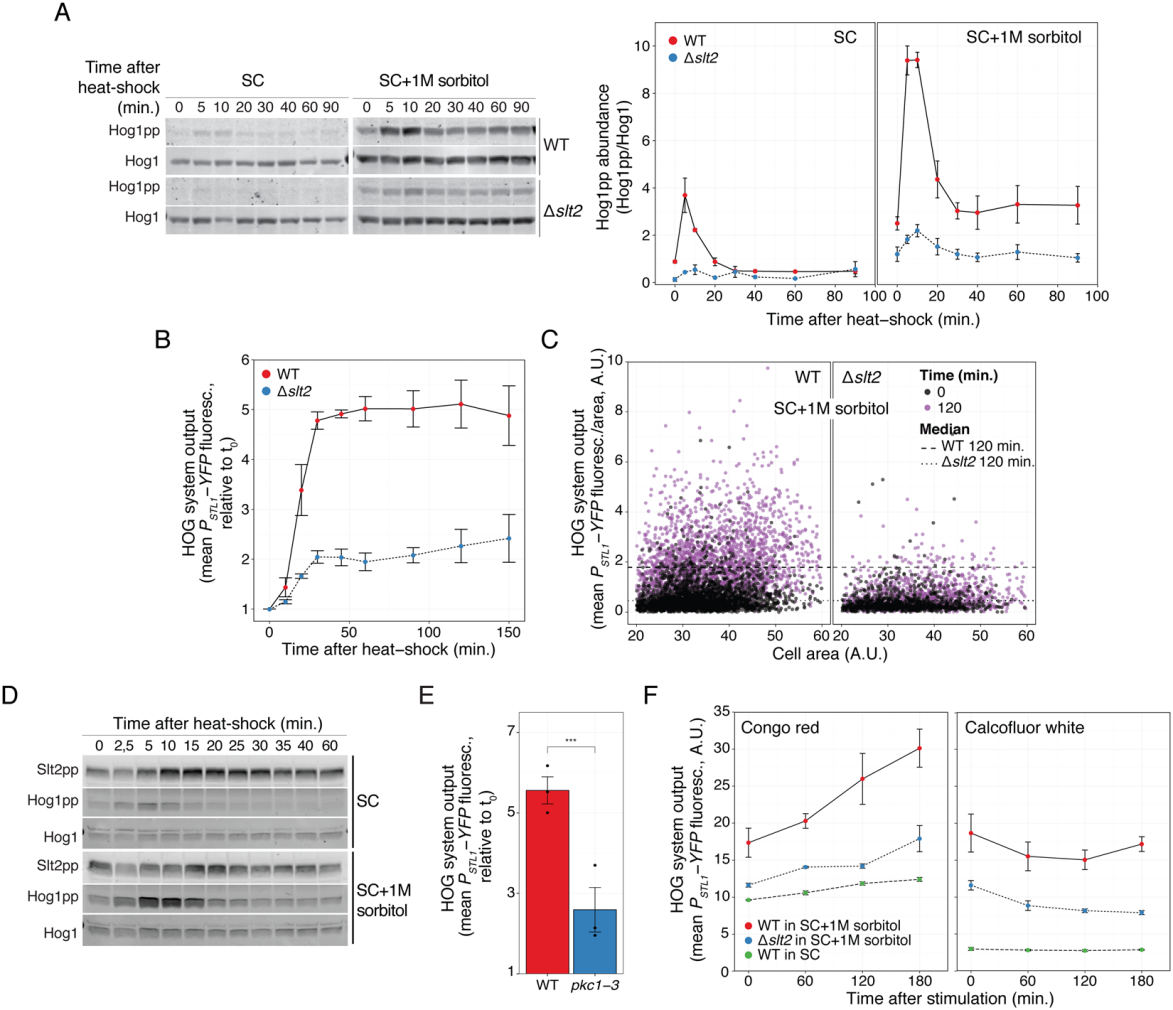
Heat-shock activation of HOG depends on the Cell-Wall Integrity pathway. (**A**) HOG MAPK activation dynamics in WT and *Δslt2* cells grown in SC or in SC + 1 M sorbitol following a shift from 30 °C to 37 °C. Left: representative blot. Uncropped blot in Fig. S3. Right. Hog1pp abundance. (**B**) HOG transcriptional reporter dynamics in WT and *Δslt2* grown in SC + 1M sorbitol following a shift from 30 °C to 37 °C. (**C**) Scatter plot comparing data at time 0 with 120 minutes after temperature shift from 30 °C to 37 °C. (**D**) CWI MAPK activation dynamics following a shift from 30 °C to 37 °C in cells grown in SC or in SC + 1 M sorbitol. Slt2 phosphorylation was assayed by immunoblotting. Uncropped blot in Fig. S8. (**E**) HOG transcriptional reporter in the temperature sensitive mutant *pkc1–3* measured 2 h after 1 M sorbitol-adapted cells were shifted from 24 °C to 37 °C. (**F**) HOG transcriptional reporter in yeast stimulated with 50 μg/ml of congo red or 50 μg/ml of calcofluor white. In A, B, E and F values correspond to the mean of three independent replicates ± SEM. Strains: LD3342, RB3376, YPD6020 and YPD6021.

Our previous work indicated a major role of the CWI MAPK cascade in HOG activation by pheromone^27^. Thus, we tested its involvement in HOG activation by heat-shock. As a first step, we measured Hog1 phosphorylation (Figs 3A and S3) and reporter induction in *Δslt2* yeast (Fig. 3B). In both measures, the response of the *Δslt2* strain was greatly reduced. Single cell analysis showed that the remaining activity observed at the population average level was not due to a small fraction of active cells (Fig. 3C). Consistent with a role of Slt2 in HOG activation, this MAPK was phosphorylated during heat-shock, both in SC and in SC supplemented with 1 M sorbitol (Figs. 3D and S8). Interestingly, Slt2 was already phosphorylated before the shock in both conditions, and it was further stimulated by heat-shock after an initial drop. Peak phosphorylation occurred between 15 and 20 minutes post stimulation, at a time in which Hog1 activation was already dropping to its lower steady-state. Nevertheless, it is noteworthy that the initial phosphorylation peak of Hog1 is diminished in *Δslt2* (both in yeast grown in SC or adapted to 1 M sorbitol in Fig. 3A), since it suggests that the CWI plays a critical role in HOG activation from the start of the heat-shock response, despite the fact that peak Slt2 phosphorylation occurs much later than Hog1’s peak. To confirm the need for Slt2 to be further activated during the heat-shock (and not just its basal activity), we used a strain with a temperature-sensitive (ts) allele of Pkc1 (*pkc1–3*)^36^, the top kinase in the CWI cascade. In this strain, the CWI pathway is functional at the permissive temperature (24 °C) and becomes quickly inactivated at 37 °C. Similar to the effect of deleting *SLT2*, induction after the temperature shift of *P*_*STL1*_*-YFP* reporter was greatly reduced in *pkc1–3* (Fig. 3E). Taken together, these results demonstrate that signaling through the CWI pathway plays a major role during the temperature shock to stimulate the HOG pathway. In addition, the residual activation of Hog1 in the absence of a working CWI reveals that there is a separate minor pathway for HOG activation.

### Congo red activates HOG via the CWI pathway

Up to this point, we have shown that two distinct inputs that cause Slt2 activation (shmoo formation during the mating response^27^ and a temperature shock) result in HOG activation. We tested two other well-known CWI stimuli, the dyes congo red (50 μg/ml) and calcofluor white (50 μg/ml), which interfere with cell wall assembly by binding to chitin^3^. At these concentrations, both drugs reduce yeast growth by 50%^37^, and caused lysis of *Δslt2* cells in SC. Similar to heat-shock and pheromone, congo red induced *P*_*STL1*_*-YFP*, and it did so in a manner dependent on high external osmolarity and the presence of the MAPK Slt2 (Fig. 3F). In contrast, calcofluor white failed to activate HOG, despite being able to activate Slt2^3^. It may be that calcofluor white has extra effects that block either HOG or the connection between Slt2 and HOG (see Discussion).

### Fps1 is essential for HOG-mediated gene expression in response to heat-shock

**Figure 4:**
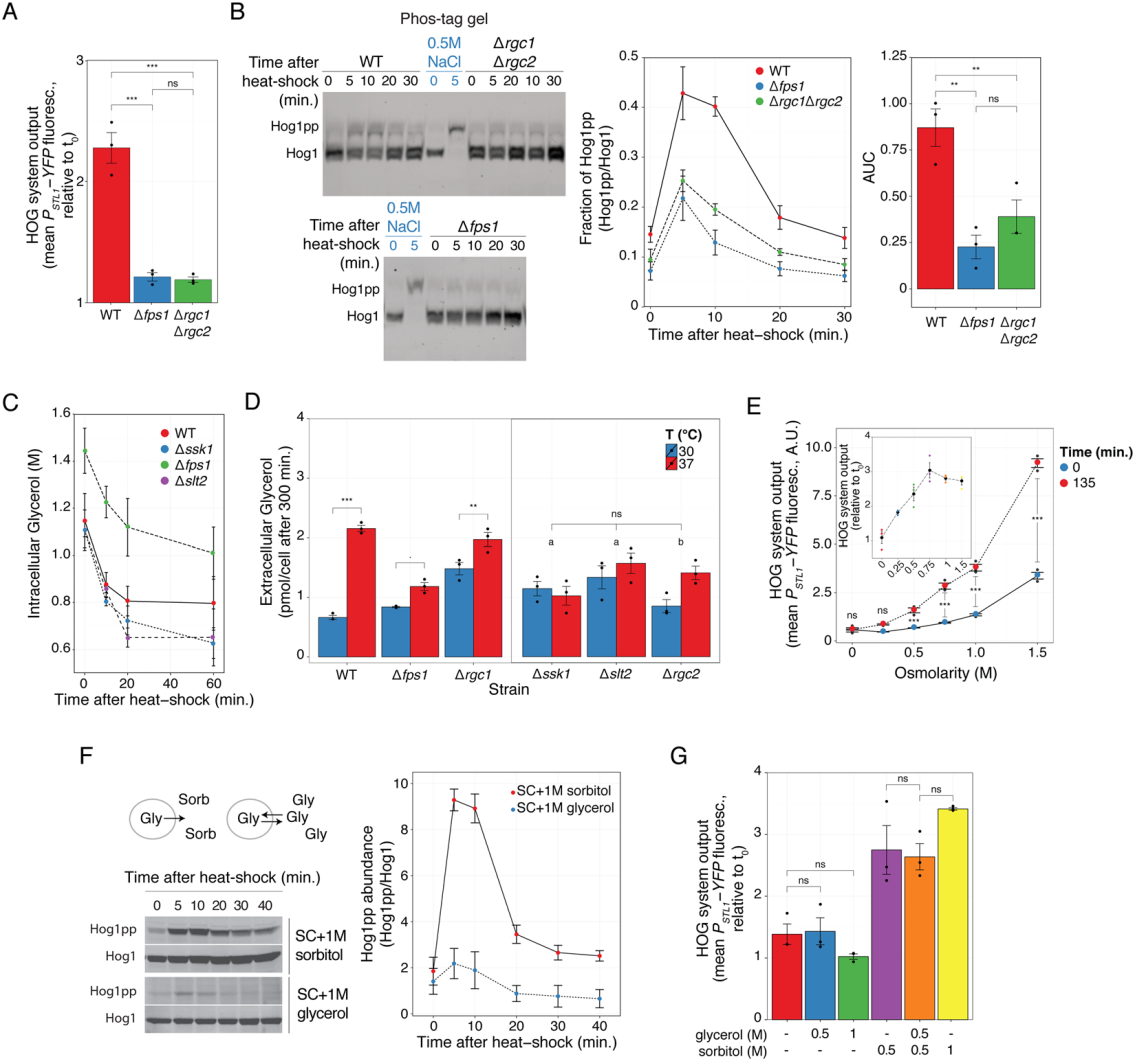
Heat-shock activation of HOG involves efflux of glycerol. (**A**) HOG transcriptional reporter in WT, *Δfps1 and Δrgc1Δrgc2* grown in SC + 1 M sorbitol measured 2 h after a shift from 30 °C to 37 °C. (**B**) Phos-tag gel showing HOG MAPK activation dynamics in WT, *Δfps1* and *Δrgc1Δrgc2* grown in SC + 1 M sorbitol following a shift from 30 °C to 37 °C, and a control of WT cells grown in SC stimulated with 0.5 M NaCl for 5 minutes. Left: representative blot. Middle. Fraction of phosphorylated Hog1. Right. Mean area under the curve (AUC) of phosphorylated Hog1 ± SEM. Uncropped blot in Fig. S11. (**C**) Intracellular glycerol measured in the indicated strains grown in 0.5 M NaCl after a shift from 26 °C to 37 °C. (**D**) Accumulated extracellular glycerol during 300 minutes of growth at 30 °C or after transfer to 37 °C of cells grown in SC + 1 M sorbitol. “a” p > 0.1 and “b” p < 0.05. (**E**) HOG transcriptional reporter in WT adapted to the indicated osmolarities at 30 °C and then transferred to 37 °C. Inset shows the increase in reporter due to the temperature shift relative to t_0_. (**F**) HOG MAPK activation dynamics in WT adapted to 1 M sorbitol or 1 M glycerol, grown at 30 °C and then transferred to 37 °C. Diagram. In medium with sorbitol, when Fps1 opens, glycerol will be lost from the cell. However, in medium with glycerol, even if Fps1 opens, the net glycerol flux should be zero. Left. Representative blot. Right. Phosphorylated Hog1 abundance. (**G**) HOG transcriptional reporter in WT adapted to the indicated mixtures of glycerol and sorbitol at 30 °C and then transferred to 37 °C. In all panels, data correspond to the mean of three independent replicates (shown as dots) ± SEM. Strains: LD3342, RB3396, RB3710, RB3717, RB3722, RB3376 and RB3382.

Our working model of HOG activation by heat-shock is centered around efflux of glycerol as the connecting link between the CWI and HOG pathways (Fig. 1B). To test the model, we deleted the genes coding for the glycerol channel, *FPS1,* or its positive regulators, *RGC1* and *RGC2*. Both in *Δfps1* and *Δrgc2*, as well as in the double knockout *Δrgc1Δrgc2*, induction of the HOG reporter was greatly reduced (Figs 4A and S9). This is consistent with an important role of glycerol loss during heat-shock in HOG activation. The single knockout *Δrgc1* showed only a partial reduction in reporter expression, indicating that this Fps1 regulator is not essential. This is interesting, since Rgc1 is required to activate HOG in response to mating pheromone^27^ (Fig. S10), an indication that the pheromone and heat-shock mechanisms of HOG activation are not identical.

### Heat-shock causes glycerol efflux

Surprisingly, Hog1 phosphorylation was not eliminated in channel deficient yeast. Phos-tag polyacrylamide gel-based western blots (Figs. 4B and S11) showed that the dynamics of Hog1 phosphorylation in WT, *Δfps1* and *Δrgc1Δrgc2* was very similar, but the peak of phospho-Hog1 was lower in the deletion strains. Total Hog1 activity over the 30-minutes experiment in *Δfps1* and *Δrgc1Δrgc2* was 25 ± 9% and 45 ± 15% of that in WT cells, respectively (Fig. 4B right). These results indicate that the initial phosphorylation peak is only partly dependent on Fps1, suggesting that glycerol release might not be necessary for Hog1 phosphorylation and/or that glycerol loss is at least partly independent of Fps1. Thus, we determined if cells actually lose glycerol after a heat-shock. To do that, we measured intracellular glycerol in WT and in strains deficient in HOG activation by this shock (*Δssk1*, *Δfps1* and *Δslt2*) adapted to 0.5 M NaCl at various times after a shift from 26 °C to 37 °C (Fig. 4C). We made two general observations: First, at time zero, *Δfps1* cells had higher glycerol content than all others, which were in turn similar to each other. This is consistent with *Δfps1* cells being less able to lose glycerol during exponential growth. Second, by 10 minutes post shift to 37 °C, there was a clear and similar drop in glycerol content in all four tested strains. By 20 minutes, intracellular glycerol concentration plateaued between 80% (*Δfps1*) and 60% (*Δslt2*) of its original glycerol content, without an apparent recovery, even after 60 minutes. The fact that *Δfps1* lost glycerol in response to the shock strongly suggested that heat-stress increases glycerol loss directly through the plasma membrane. Phospholipid bilayers do become more permeable to small molecules like glucose and glycerol with increasing temperatures^38^, so this is not unexpected. It was interesting though that the drop in *Δfps1* was similar to WT, since it indicated that Fps1 aperture might not take place immediately, or that initial loss via the membrane is large enough to mask the contribution of efflux via the glycerol channel.

Yeast adapt the lipid composition of their membranes to counteract changes in its fluidity caused by temperature^39^, and that might affect glycerol permeability. Also, an increased glycerol metabolism at 37 °C might contribute to the drop in intracellular glycerol. Consequently, the role of glycerol efflux in general, and via Fps1 in particular, might be better assessed measuring its extracellular accumulation at longer times. For these reasons, we measured extracellular accumulation of glycerol after five hours post shift from 30 °C to 37 °C in the strains used above as well as in *Δrgc1* and *Δrgc2*. Extracellular content reflects the combined ability of cells to release as well as to synthesize and metabolize glycerol. The supernatant of WT cells subjected to heat-shock contained more glycerol than without the temperature shift (Fig. 4D). In contrast, the effect of temperature shift was absent (*Δslt2* and *Δssk1*) or greatly reduced (*Δfps1 and Δrgc2*) in the strains that fail to activate HOG. That *Δfps1* did show some increase in the accumulated glycerol in the extracellular media confirmed that an alternative route for glycerol efflux is stimulated by the temperature shift, likely by increasing plasma membrane permeability. The strain that had the largest residual ability to release glycerol at 37 °C, *Δrgc1*, is the strain that showed only a partial defect in HOG reporter induction by heat-stress, supporting the link between glycerol efflux and HOG activation. According to our model, *Δssk1* cells should not show any defect in Fps1 aperture upon heat-shock. Thus, the failure of this strain to accumulate extracellular glycerol perhaps reflects its inability to activate HOG and in this way boost glycerol production.

### Glycerol efflux is required for heat-shock activation of HOG

To provide more evidence that glycerol efflux controls HOG activation by heat-shock, we altered the gradient driving glycerol out. We did this in two ways. First, we adapted yeast to growth in medium with different osmolarities. At higher external osmolarities, cells need to accumulate glycerol to higher concentrations to prevent water loss and thus maintain turgor. Since heat-shock causes glycerol loss (Figs. 4C and D), cells will lose more at higher osmolarities (due to a larger chemical gradient), turgor will be reduced more, and HOG should be activated to a greater extent. Indeed, heat-shock induced the HOG reporter more strongly at higher osmolarities (Fig. 4E) while a control reporter was not affected (Fig. S12). Although accumulation of reporter did not reach an absolute maximum at the highest osmolarity we tested (1.5 M sorbitol), fold-change induction plateaued at 0.75 M sorbitol (Fig. 4E, inset).

Second, we replaced the added sorbitol with glycerol, eliminating in this way the concentration gradient driving glycerol out, but maintaining the high external osmolarity. (Note that glycerol, used as an osmotic stressor, is able to activate HOG Fig. S13). Thus, even if cells open Fps1, the net glycerol flux should be zero. This change led to minimal Hog1 phosphorylation by heat-shock, to the same low levels observed in SC (Figs. 4F and S14) and abolished reporter induction (Figs. 4G and S15). We also tested a 0.5 M sorbitol-0.5 M glycerol mix, which has the same external osmolarity as 1 M sorbitol but a chemical gradient equivalent to just 0.5 M sorbitol. In this mix, HOG activation reached levels similar to just 0.5 M sorbitol. These results support the role of glycerol loss as the driver for HOG activation by heat-shock.

**Figure 5:**
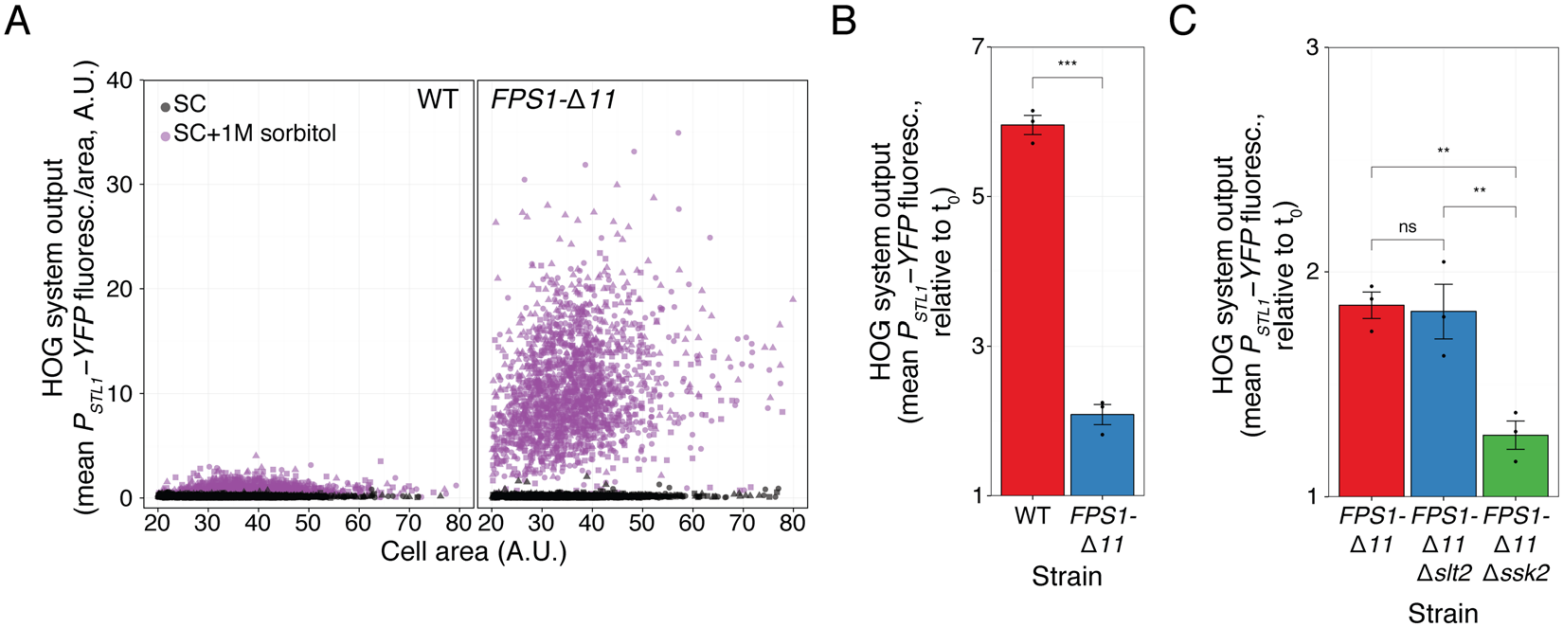
Constitutively open Fps1 bypasses the need of the CWI pathway. (**A**) HOG transcriptional reporter comparing WT and *FPS1-Δ11* grown in SC or adapted to SC + 1 M sorbitol . Scatter plot shows HOG system output/area vs. cell area. (**B**) HOG transcriptional reporter in WT and *FPS1-Δ11* grown in SC + 1 M sorbitol measured 2 h after a shift from 24 °C to 37 °C. Values correspond to the mean of three independent replicates (shown as points) ± SEM, relative to t_0_. (**C**) Same as in B for *FPS1-Δ11,* *FPS1-Δ11* *Δslt2* and *FPS1-Δ11* *Δssk2*. Strains: LD3342, YPD6023, PAY6024 and PAY6025.

Fps1-independent glycerol efflux helps explain why Hog1 is phosphorylated in *Δfps1* cells (Fig. 4B), but it does not explain why this strain failed to activate the reporter gene. We suggest that the initial phosphorylation peak might not be enough to induce gene expression and that sustained glycerol efflux via Fps1 is necessary. This may be accomplished using a constitutively open Fps1 mutant. If opening of Fps1 is a major cause for HOG activation by heat-stock, then a strain expressing this mutant should be largely insensitive to a temperature shift. To test that idea, we replaced *FPS1* by *FPS1-Δ11*, which bears a short deletion between amino acids 216 and 231 in the channel’s regulatory domain^40^. This strain exhibited a high basal expression of *P*_*STL1*_*-YFP* reporter when grown in 1 M sorbitol (approximately 18-fold higher than *FPS1* cells) (Fig. 5A), but not on SC. It also displayed a strong response, with no apparent adaptation, to a 0.5 M NaCl shock (Fig. S16). These behaviors are consistent with the expected inability of *FPS1-Δ11* cells to retain glycerol, forcing the cells to maintain an active HOG pathway to boost glycerol production. When this strain, grown in 1 M sorbitol, was shifted from 24 °C to 37 °C, reporter expression was further induced (Fig. 5B), but to a lower degree than *FPS1* (6-fold increase in *FPS1* vs. 2-fold in *FPS1-Δ11*). This suggests that heat-shock stimulates HOG in large part, but not exclusively, by opening Fps1. As expected, stimulation of the HOG reporter in *FPS1-Δ11* yeast was absent in the Sln1 branch MAPKKK mutant *Δssk2* (Fig. 5C), confirming once more the absolute need of HOG activation for gene expression. Interestingly, the residual activation in *FPS1-Δ11* was unaffected by deletion of Slt2 (Fig. 5C). Thus, when cells express a form of Fps1 whose aperture cannot be regulated, HOG activation becomes independent of the CWI pathway. This result strongly suggests that the main role of Slt2 is to open Fps1.

## Discussion

**Figure 6:**
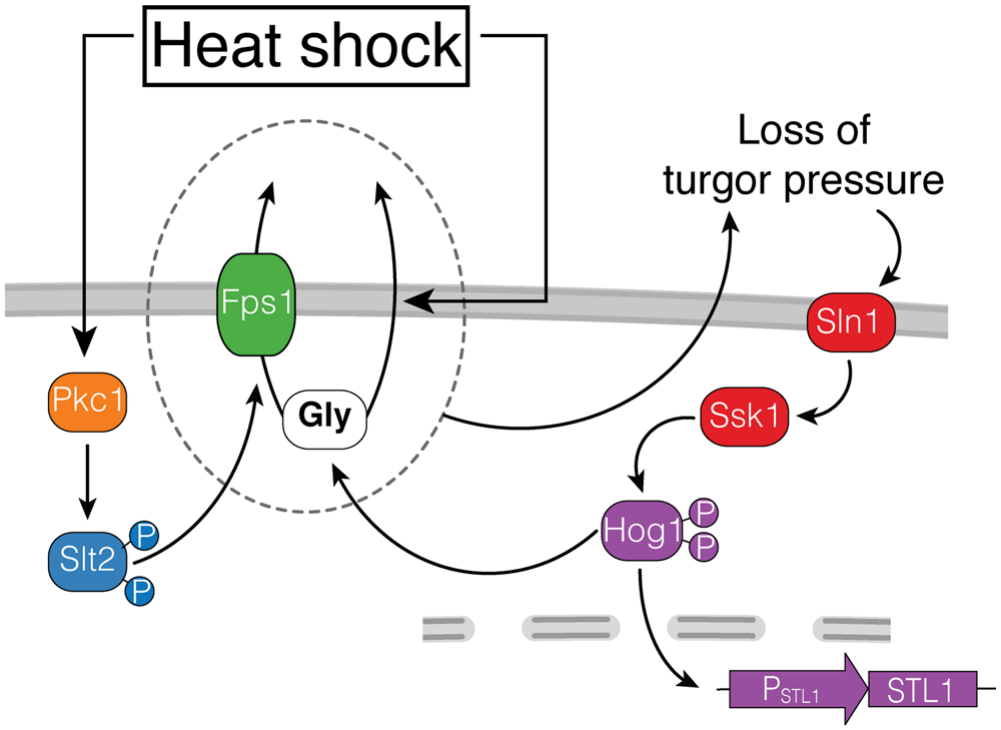
Model of HOG activation by heat-shock. Heat-shock stimulates glycerol efflux by at least two paths. One, independent of the glycerolporin Fps1, might be directly through the phospholipid bilayer. The other, Fps1-dependent, requires the activation of the CWI Pkc1-Slt2 pathway. Efflux increases when there is a large chemical gradient driving glycerol out, which happens when yeast grow in high osmolarity medium. Glycerol efflux causes reduced turgor pressure what in turn stimulates the Sln1 branch (Ssk1) of HOG. Activated Hog1 induces gene expression (*P*_*STL1*_*-YFP*) and glycerol synthesis, which maintains a high chemical gradient, closing the circle.

Here, we have shown that stimulation of the CWI pathway causes HOG activation. Our data is consistent with the mechanism of activation involving the release of glycerol in a manner largely dependent on the MAPK Slt2 (Fig. 6). The exit of glycerol is accompanied by water and thus causes loss of turgor pressure, leading to the activation of the HOG pathway. Activated Hog1 stimulates glycerol production so that cells can reestablish their turgor pressure. Continued glycerol efflux through Fps1 maintains HOG active and induces HOG-dependent transcription. The entire process is amplified when yeast grow in high external osmolarity, since the gradient driving glycerol out is greater under such conditions.

Upon heat-stress, Hog1 is phosphorylated following a “peak and decline to a lower plateau” dynamics. In our W303-1A genetic background, phosphorylation was dependent on the Sln1 branch, both in SC and in high osmolarity medium. This is in contrast to a previous report that showed exclusive dependence on the Sho1 branch, at least in SC^10^. We have no explanation for this discrepancy other than possible differences between DF5 and W303 genetic backgrounds.

Our data indicates that strong Hog1 phosphorylation depends on high external osmolarity, on glycerol gradient and on the activity of Slt2. In contrast, the shape of its dynamics is independent of all of them, since a small transient peak is still detectable in *Δslt2* yeast grown in SC supplemented with 1 M sorbitol and WT grown in SC. The effect of external osmolarity may be safely attributed to the amplitude of the glycerol gradient, and not to external osmolarity *per se*, since using glycerol instead of sorbitol reduces Hog1 phosphorylation to the values observed in SC. Initially, we hypothesized that the role of Slt2 on Hog1 phosphorylation could be completely explained by assuming that Slt2 causes Fps1 aperture, triggering the above explained activation of the Sln1 branch. This idea was further supported by our observation that Slt2 was required for the heat-shock-dependent increase in accumulated extracellular glycerol and by the fact that HOG activity becomes independent of Slt2 in *FPS1-Δ11* yeast, where the channel is constitutively open and unregulated. However, the observation that in *Δfps1* yeast, peak phosphorylation of Hog1 was not abolished but reduced, indicated that Slt2 regulates Hog1 phosphorylation during heat-shock also by another mechanism, perhaps by inhibiting HOG pathway phosphatases, such as Ptp2, Ptp3 or Ptc1,2,3, all of which can target Hog1^41^. Such inhibition might be important, given that the HOG pathway has a significant basal activity, even in SC^42^, which is further increased in yeast adapted to high osmolarity (evident at time zero in our western blots and reporter assays (Fig. 1F, G and C)). The experiments shown in Figs. 5B and C suggest a second minor mechanism for HOG activation by heat-shock, independent of both Fps1 and Slt2.

The behavior of *Δfps1* strain was surprising. This strain cannot stimulate *P*_*STL1*_*-YFP* expression in response to heat-shock, even though it exhibits robust Hog1 phosphorylation. This decoupling of phosphorylation and transcription has been described previously in the response to arsenite or antimony stress^8^, although no mechanism was suggested. On the one hand, it is possible that the lower phosphorylation and somewhat faster drop in phosphorylated Hog1 in *Δfps1* cells could be enough to prevent Hog1 dependent transcription. This is what happens in many cells exposed to a small hyperosmotic shock, where Hog1 returns to the cytoplasm before the *STL1* locus is induced^33^. Alternatively, Fps1 might serve a second function, unrelated to glycerol release, required for Hog1 to stimulate transcription in this context. A more likely explanation is that loss of glycerol through the plasma membrane is the main cause of Hog1 phosphorylation in *Δfps1*, and that after a period of time, the plasma membrane could modify its composition in order to diminish glycerol permeability, which would turn off the stimulus responsible for HOG activation. Continued loss of glycerol through Fps1, in this scenario, would be responsible for the sustained HOG-dependent gene induction.

We have stimulated the CWI MAPK Slt2 with four different external stimuli (mating pheromone^27^, heat-stress, congo red and calcofluor white). All but calcofluor white stimulated the HOG pathway and all in a manner that depended on Slt2. Given that calcofluor white activates Slt2, the inability of this drug to activate HOG suggested it might have an extra effect that prevented *P*_*STL1*_*-YFP* induction. Global gene expression analysis provides some support for this idea, since the two drugs induce only partly overlapping gene expression profiles^37^. In agreement with our result, Talemi *et al*.^43^ also found that calcofluor white does not cause Hog1 phosphorylation. The other possibility, namely that congo red has an extra effect necessary to activate HOG, is less likely, since the two other CWI stimuli we used (mating pheromone and temperature) did activate HOG.

Here we have shown that heat-shock activates two MAPK cascades that usually act in opposing osmotic responses. The net result is a high turnover rate, with constant loss and synthesis of glycerol. Why so much energy waste? There could be many scenarios possible. First, one natural micro-environment where yeast grow is the surface of a fruit, with a thin layer of water. During daylight, high radiation could lead to increased temperature and consequently high water evaporation. If heat induces yeast to stimulate glycerol release, thanks to its *humidity stabilizing* property (augments the boiling point of water), its increased external concentration will help reduce evaporation. During the cold of night time, yeast would also benefit from high external glycerol, since this polyol is also a cryo-protectant.

Another role for the high glycerol turnover rate system could be to minimize oxidative stress due to reactive oxygen species (ROS) generated during respiration. Respiration is necessary to regenerate the NAD^+^ consumed during glycolysis but has the drawback of generating ROS. Another way to regenerate NAD^+^ is fermentation. However, its product, ethanol, is another source of stress due to its deleterious effects including membrane permeabilization, cytosolic acidification, protein denaturation and again, ROS generation^44^. A third way to regenerate NAD^+^ is glycerol production. Glycerol-3-phosphate production by the NAD-dependent glycerol-3-phosphate dehydrogenase (Gpd1 and 2) leads to NAD^+^ release. Thus, stimulating its efflux increases NAD^+^ regeneration by this route, without the complications of excessive respiration or fermentation.

Fps1 has 13 homologs in mammals, aquaporins AQP0-12, some of which, like Fps1, also transport glycerol (AQP3,7,9 and 10)^45^. They have been reported to function in diverse processes, including energy metabolism, cancer and wound healing^45–47^. As we proposed above for yeast, glycerol is a natural moisturizer in mammals, in this case produced by the skin^48^. Skin keratinocytes express the aquaglyceroporin *AQP3*, which is involved in delivering glycerol to the stratus corneum (the most external layer of the epidermis). AQP3 null mice have decreased glycerol permeability, leading to *dry-skin* and reduced hydration^49^. Glycerol replacement was found to correct hydration. Thus, it appears that this function is similar to our proposal above for yeast: increase the hydration of an extracellular thin layer. Notably, AQPs expressed in the basolateral membrane of many epithelia, seem to play a role in cancer^45,47^. Although the mechanism that links AQPs to cell proliferation and invasion is still not clear, it is tempting to speculate that, as in yeast, regulation of glycerol turnover might be involved, for example, in stimulating MAPK activity.

In conclusion, we found that heat-shock robustly stimulates HOG in a largely indirect fashion, via the CWI. We suggest that Fps1 acts as a key regulatory node whereby different MAPK cascades converge to jointly adjust stress responses. The net stimulation of HOG by heat-shock varies depending on the actual environmental conditions, from low to high in accordance with the level of external osmolarity. The wealth of accumulated knowledge about MAPK cascades in yeast offers the opportunity to study how these networks of signaling evolve and to generate hypothesis and working models as to how similar networks operate in higher eukaryotes.

## Materials and Methods

### Genetic and molecular biological methods

All nucleic acid manipulations and yeast methods were done as described^50^.

### Yeast strains

Strains used in the paper appear in Table 1 and oligos in Table 2. We made deletions by PCR, as described^51^.

**Table 1:** Yeast strains.

Strain	Genetic Background	Relevant genotype	Reference
ACL3341	*W303*	*MATa bar1Δ prm1::P*_*PRM1*_ *-mCherry::hygB STL1::P*_*STL1*_ *-YFP::URA3*	^27^
LD3342	*W303*	*MATa bar1Δ prm1::P*_*PRM1*_ *-mCherry::hygB STL1::P*_*STL1*_ *-YFP::URA3 BMH2::P*_*BMH2*_ *-CFP::TRP1*	^27^
RB3376	*W303*	*MATa bar1Δ slt2Δ::kanMX6 prm1::P*_*PRM1*_ *-mCherry::hygB STL1::P*_*STL1*_ *-YFP::URA3 BMH2::P*_*BMH2*_ *-CFP::TRP1*	^27^
RB3382	*W303*	*MATa bar1Δ ssk1Δ::kanMX6 prm1::P*_*PRM1*_ *-mCherry::hygB STL1::P*_*STL1*_ *-YFP::URA3 BMH2::P*_*BMH2*_ *-CFP::TRP1*	^27^
RB3642	*W303*	*MATa bar1Δ ssk2Δ::his3MX6 prm1::P*_*PRM1*_ *-mCherry::hygB STL1::P*_*STL1*_ *-YFP::URA3 BMH2::P*_*BMH2*_ *-CFP::TRP1*	^27^
RB3691	*W303*	*MATa bar1Δ ssk22Δ::kanMX6 prm1::P*_*PRM1*_ *-mCherry::hygB STL1::P*_*STL1*_ *-YFP::URA3 BMH2::P*_*BMH2*_ *-CFP::TRP1*	^27^
RB3695	*W303*	*MATa bar1Δ ssk2Δ::his3MX6 ssk22Δ::kanMX6 prm1::P*_*PRM1*_ *-mCherry::hygB STL1::P*_*STL1*_ *-YFP::URA3 BMH2::P*_*BMH2*_ *-CFP::TRP1*	This study
RB3396	*W303*	*MATa bar1Δ fps1Δ::his3MX6 prm1::P*_*PRM1*_ *-mCherry::hygB STL1::P*_*STL1*_ *-YFP::URA3 BMH2::P*_*BMH2*_ *-CFP::TRP1*	^27^
RB3704	*W303*	*MATa bar1Δ sho1Δ::kanMX6 prm1::P*_*PRM1*_ *-mCherry::hygB STL1::P*_*STL1*_ *-YFP::URA3 BMH2::P*_*BMH2*_ *-CFP::TRP1*	^27^
RB3710	*W303*	*MATa bar1Δ rgc1Δ::kanMX6 prm1::P*_*PRM1*_ *-mCherry::hygB STL1::P*_*STL1*_ *-YFP::URA3 BMH2::P*_*BMH2*_ *-CFP::TRP1*	^27^
RB3717	*W303*	*MATa bar1Δ rgc2Δ::kanMX6 prm1::P*_*PRM1*_ *-mCherry::hygB STL1::P*_*STL1*_ *-YFP::URA3 BMH2::P*_*BMH2*_ *-CFP::TRP1*	^27^
RB3722	*W303*	*MATa bar1Δ rgc1Δ::kanMX6 rgc2Δ::his3MX6 prm1::P*_*PRM1*_ *-mCherry::hygB STL1::P*_*STL1*_ *-YFP::URA3 BMH2::P*_*BMH2*_ *-CFP::TRP1*	^27^
YRB3801	*W303*	*MATa bar1Δ hog1Δ::kanMX6 prm1::P*_*PRM1*_ *-mCherry::hygB STL1::P*_*STL1*_ *-YFP::URA3 BMH2::P*_*BMH2*_ *-CFP::TRP1*	^27^
RB3937	*W303*	*MATa bar1Δ hog1::hog1T100A prm1::P*_*PRM1*_ *-mCherry::hygB STL1::P*_*STL1*_ *-YFP::URA3 BMH2::P*_*BMH2*_ *-CFP::TRP1*	This study
RB3938	*W303*	*MATa bar1Δ hog1::hog1T100M prm1::P*_*PRM1*_ *-mCherry::hygB STL1::P*_*STL1*_ *-YFP::URA3 BMH2::P*_*BMH2*_ *-CFP::TRP1*	This study
YPD5937	*W303*	*MATa bar1Δ sho1Δ::natMX ssk1Δ::kanMX6 prm1::P*_*PRM1*_ *-mCherry::hygB STL1::P*_*STL1*_ *-YFP::URA3 BMH2::P*_*BMH2*_ *-CFP::TRP1*	This study
YGB5938	*W303*	*MATa bar1Δ prm1::P*_*PRM1*_ *-mCherry::hygB STL1::P*_*STL1*_ *-YFP::URA3 STL1::P*_*STL1*_ *-tdTomato::LEU2*	This study
YPD6020	*S288C*	*MATa ura10Δ0::kanMX4 his3Δ1 leu2Δ0 ura3Δ0 met15Δ0 P*_*STL1*_ *-YFP-CEN-LEU2*	This study
YPD6021	*S288C*	*MATa pkc1–3::kanMX4 his3Δ1 leu2Δ0 ura3Δ0 met15Δ0 P*_*STL1*_ *-YFP-CEN-LEU2*	This study
YPD6022	*W303*	*MATa bar1Δ STL1::P*_*STL1*_ *-YFP::TRP1*	This study
YPD6023	*W303*	*MATa bar1Δ FPS1::FPS1-Δ11 STL1::P*_*STL1*_ *-YFP::TRP1*	This study
PAY6024	*W303*	*MATa bar1Δ ssk2Δ::his3MX6 FPS1::FPS1-Δ11 STL1::P*_*STL1*_ *-YFP::TRP1*	This study
PAY6025	*W303*	*MATa bar1Δ slt2Δ::his3MX6 FPS1::FPS1-Δ11 STL1::P*_*STL1*_ *-YFP::TRP1*	This study

**Table 2:** Oligos.

Name	Sequence
oHog1-prom-fw	*ACCTCAAAGCGCTTCGTCATGG*
oHog1-term-rev	*TATTTATGAAAATTCCTCTTCGG*
oHog1-TA-direct	*TGGAAGATATATATTTTGTC* ***GCT*** *GAATTACAAGGAACAGATTTACATAGAC*
oHog1-TA-reverse	*GTAAATCTGTTCCTTGTAATTC* ***AGC*** *GACAAAATATATATCTTCCAATGGAGAAAG*
oHog1-TM-direct	*TGGAAGATATATATTTTGTC* ***ATG*** *GAATTACAAGGAACAGATTTACATAGAC*
oHog1-TM-reverse	*GTAAATCTGTTCCTTGTAATTC* ***CAT*** *GACAAAATATATATCTTCCAATGGAGAAAG*
5′del_Fps1–418	*CCAAACTAATATGGAAAGCAATGAATCACCACGTAACGTCgatctgtttagcttgcctcg*
3′del_Fps1–1611	*TAATCATTTCCTTATAAACTGGTAAAGACCAGTTGACTGGgcaggttaacctggcttatc*
5′Fps1-Δ11	*CCAAACTAATATGGAAAGCAATGAATCACCACGTAACGTCacacctacagtcttgccctc*
3′Fps1_Δ11	*CTTATAAACTGGTAAAGACCAGTTGAC*
Rv_sgRNA_URA3_C.alb_CRISPR	*TAGCTCTAAAACttaatcgaaataatcgctgtGATCATTTATCTTTCACTGCGGA*
Fw_sgRNA_URA3_C.alb_CRISPR	*TCacagcgattatttcgattaaGTTTTAGAGCTAGAAATAGCAAGTTAAA*

P_STL1_-YFP reporter strains, except those indicated below, derive from LD3342^27^, which in turn derived from ACL379 (*can1::P*_*HO*_*-CAN1 ho::P*_*HO*_*-ADE2 bar1Δ ura3 ade2 leu2 trp1 his3*)^35^, a W303-1A descendant. The strain has three reporters: a HOG-inducible reporter (*P*_*STL1*_*-YFP*) inserted in the *STL1* promoter; a mating pheromone-inducible reporter (*P*_*PRM1*_*-mCherry*); and a constitutive reporter, *P*_*BMH2*_*-CFP*, in *BHM2* promoter.

We made *P*_*STL1*_*-tdTomato* reporter strain inserting plasmid pJT3557^52^, a Yiplac128 bearing *P*_*STL1*_*-tdTomato*, in *STL1* locus (by cutting it with NruI) into strain ACL3341, and selecting in -leucine medium.

We made strains YPD6020 and YPD6021 by transforming *P*_*STL1*_*-YFP-CEN-LEU2* into WT dma3865 and *pkc1–3* tsa541 strains^53^.

We made strain *FPS1-Δ*11 as follows. First, we truncated *FPS1* in ACL379 by inserting a PCR product containing the *P*_*TEF*_*-caURA3-T*_*TEF*_ cassette replacing nucleotides +649 and +1610 by homologous recombination using oligos 5′del_Fps1-418 and 3′del_Fps1-1611. Second, we transformed the resulting *fps1* truncated strain with plasmid *p414-TEF1p-Cas9-CYC1t* (Addgene #43802) and selected in tryptophan minus plates. Third, we transformed this strain with a mix of a plasmid *sgRNA_URA3MX_C*.*alb_CRISPR* that expresses a guide for Cas9 directed to the *C*. *albicans URA3* together and a PCR product made with oligos 5′Fps1-Δ11 and 3′Fps1-Δ11 that contained the *FPS1* sequence to recombine out *URA3* and at the same time bearing the desired deletion of nucleotides corresponding to amino acids 216 and 231 of *FPS1*, to create the *FPS1-Δ*11 mutation. We verified the mutation by sequencing. Finally, we created strain YPD6023 inserting the *P*_*STL1*_*-YFP-404* reporter plasmid cut with NruI.

To make the *HOG1-T100A* and *HOG1-T100M* containing strains, we made a PCR fragment covering the entire *HOG1* ORF with the T100A or T100M mutation in two steps. First, we generated two PCR fragments, using yeast genomic DNA as template, that overlap at the location of the desired mutation and the oligos as follows:

For Hog1-T100A (Hog1-T100M), we generated fragments using *oHog1-prom-fw* and *oHog1-TA-reverse (oHog1-TM-reverse)*, and *oHog1-term-rev* with *oHog1-TA-direct (oHog1-TM-direct)*. We then performed a second PCR with the external oligos *oHog1-prom-fw* and *oHog1-term-rev* using the two PCR products generated in the first step for each mutation as template. Finally, we transformed this PCR product into a *Δhog1::kanMX6* strain, YRB3801^27^, and selected transformants in YPD plates supplemented with 0.5 M NaCl (in which the parental *Δhog1* does not grow). We confirmed the resulting strains by sequencing.

### Plasmids

To make P_STL1_-YFP-404 we subcloned YFP into P_STL1_-CFP-404^27^.

To make *P*_*STL1*_*-YFP-CEN-LEU2*, we co-transformed plasmid P_STL1_-YFP-404 cut with AatII and SnaBI together and plasmid pRS415 cut with NotI into ACL379 and selected in leucine minus plates. We then recovered the plasmid.

To make *sgRNA_URA3MX_C*.*alb_CRISPR* in two steps. First, we made two overlapping PCR products using a T7 primer with Rv_sgRNA_URA3_C.alb_CRISPR and T3 primer with Fw_sgRNA_URA3_C.alb_CRISPR using *p426-SNR52p-gRNA*.*CAN1*.*Y-SUP4* as template (Addgene#43803). Then, using isothermal assembly^54^ we connected these two products together with plasmid pRG225 (Addgene #64526).

### Single-cell microscopy methods

We cultured yeast in SC medium supplemented or not with NaCl, sorbitol and/or glycerol and allowed them to grow to exponential phase for at least 15 hs at 24 °C, 26 °C or 30 °C with agitation. We used cultures in exponential growth (A_600nm_ between 0.05 and 0.3) to minimize cellular autofluorescence, especially in the vacuole^35,55^. We took samples for microscopy and added cycloheximide (50 μg/ml) to stop further translation and allow full maturation of the reporter fluorescent proteins for at least 3 hs at room temperature or 15 hs at 4 °C before imaging^35^.

We imaged using an oil-immersion 60x PlanApo objective (NA = 1.4) in an Olympus IX81 inverted microscope equipped with CoolLED illumination, a motorized XYZ stage and a CoolSnapHQ2 cooled charge-coupled device camera (Photometrix) in a 30 °C room. We used MetaMorph 7.5 software (Universal Imaging Corporation) to image and control the microscope. We acquired bright-field, mCherry or tdTomato, YFP, and/or CFP fluorescence images using filter sets 41004, 31044v2 and 41028 from Chroma Technologies Corp.

To extract quantitative information, we processed images with Cell-ID as previously described^55,56^, and analyzed it with R (http://www.r-project.org) and the package Rcell (https://cran.r-project.org/src/contrib/Archive/Rcell/).

To calculate the transcriptional output (defined as the total corrected fluorescence signal in a cell from the corresponding reporter gene), we subtracted the background signal measured outside cells (the mode of the distribution of all fluorescence pixels not associated with any cell) from the integrated total fluorescence for a given cell (the sum of the fluorescence values of all the pixels that Cell-ID associated with that cell). When indicated, we normalized this value to the area of the cells.

### Analysis of cell-to-cell variability

We performed the analysis as in Colman-Lerner *et al*.^35^. We measured total variability as η^2^_tot_ STD^2^_(xFP)/<xFP>_; η^2^_int_, gene expression noise (“intrinsic noise”^34^), as the variance in the difference of the normalized abundance of the two fluorescent proteins driven by identical copies of *P*_*STL1*_, divided by two (0.5 x STD^2^_(YFP/<YFP> - tdTomato/<tdTomato>)_); and η^2^_ext_, extrinsic noise, as the difference between η^2^_tot_ and η^2^_int_.

We estimated the correlation ρ using the cor() function of R (Pearson method).

### Protein methods

We prepared samples essentially as described^57^. Briefly, for each time point, we mixed 2 ml of exponentially growing cells (A_600nm_ ≤ 0.6) with 165 μL of 10 M NaOH and 20 μL of 100% 2-mercaptoethanol (premixed immediately before the experiment). We then incubated samples at room temperature for at least 5 min, then we added 300 μl of 100% trichloroacetic acid (Sigma-Aldrich), followed by 10 minutes on ice and centrifugation at 16000 g for 2 minutes at 4 °C. We washed the pellet with 900 μl of an ice-cold acetone solution (70% (v/v) acetone in 100 mM tris-HCl, pH 6.8) by vortexing, followed by centrifugation. We air-dried the pellet and resuspended it in 50 μl of resuspension buffer (3% SDS, 100 mM tris-HCl, pH 6.8) for 30 min, switching between heating at 65 °C and a Genie disruptor (Scientific Industries) (total time of disruptor = 10 min). Finally, we centrifuged samples for 10 minutes at 16000 g (at 4 °C) to clear cellular debris and stored 40 μl of the supernatants at −80 °C, until used in conventional SDS-PAGE or phos-tag gels.

For gel loading, we mixed 40 μl of protein solutions with 10 μl of SDS–polyacrylamide gel electrophoresis loading buffer 5X (25% 2-mercaptoethanol, 50% glycerol, 10% SDS, 150 mM tris-HCl (pH 6.8), 0.002% bromophenol blue), boiled for 5 min, placed on ice and loaded on the gel.

We used 12% tris-glycine polyacrylamide gels (12% acrylamide, tris-HCl 375 mM (pH 8.8), 0,1% SDS, 0,1% APS, 0,004% TEMED), then transferred onto a 0.45-μm Amersham Hybond-ECL nitrocellulose membrane (RPN303D, GE Healthcare). We blocked membranes for at least 1 h at room temperature in blocking buffer solution T-TBS (TBS 1X−20 mM tris 140 mM NaCl (pH 7.4)-, milk 5%, tween-20 0.05%). We detected phosphorylated MAPK Mpk1/Slt2 with a rabbit anti–phospho-p44/p42 at 1:1000 dilution (Cell Signaling Technology, #9101 L), pp-Hog1 with a mouse anti–phospho-p38 at 1:1000 dilution (Cell Signaling Technology, #9216 L) and total Hog1 with a goat anti-Hog1 (#SC-6815) at 1:1000 dilution (Santa Cruz Biotechnology Inc.) in blocking buffer solution.

We washed in TBS with 0.05% tween-20 and probed for 1 h at room temperature with a donkey anti-rabbit (#926-32213 IRDye 800CW), a donkey anti-mouse (#926-32212 IRDye 800CW), a donkey anti-goat (#926-68074 IRDye 680RD) and a donkey anti-rabbit (#926-68073 IRDye 680RD) fluorophore-conjugated secondary antibodies (Li-Cor Biosciences), all diluted 1:10,000 in blocking buffer solution.

Finally, we washed blots 3 times in TBS with 0.05% tween-20 and once in TBS and then scanned them on a Li-Cor infrared imaging system (Li-Cor Biosciences). We quantified band intensities with the Odyssey Image Studio software (Li-Cor Biosciences) using the median intensity of pixels in a border around a shape for background correction.

For phos-tag gel electrophoresis, we used 8% polyacrylamide with 20 μM phos-tag (Wako Chemical Industries #304-93521) following the protocol described previously^31^, except for the transfer buffer: we used tris-glycine, methanol, as described for conventional PAGE.

### Glycerol measurements

Extracellular glycerol was measured essentially as described^27^. Yeast cells were grown overnight in SC medium supplemented with 1 M sorbitol (SCS) at 30 °C. For this we started with a diluted culture to obtain an A_600nm_ of 0.2 the following day. Next day, the culture was filtered (cellulose ester filtering discs of 0.45 μm pore size and 25 mm diameter; Millipore, catalog no. HAWP02500) in a “1225 manifold” (Millipore, catalog no. XX2702550) and resuspended in fresh SCS medium. This step is necessary to remove glycerol accumulated before the experiment.

Next, we split this culture into two flasks, one for the 37 °C heat-shock and the other for 30 °C control. After 300 minutes we filtered 5 mL of cells (HAWP02500, Millipore filtered) and collected the flow through in 15 mL falcon tubes. Finally, the concentration of glycerol was measured by high-pH anion exchange chromatography with pulse amperometric detection in an ICS-3000 chromatographic system (Dionex), as previously described^58^. We used a CarboPac MA1 column (4 × 250 mm, Dionex) and a CarboPac MA1 guard column (4 × 50 mm, Dionex). An isocratic program with 200 mM NaOH was used at a flow rate of 0.5 ml/min and a loop of 20 ml. The standard curve was measured between 0 and 4000 ng of glycerol. Samples were diluted as necessary to obtain data in this range.

The following mathematical transformation was used to calculate the amount of glycerol produced *per cell*:

$${\tfrac{Glycerol(pmols)}{cell}|}_{tx}={\tfrac{{[Glycerol]}_{tx}(ng/\mu L)}{O{D}_{tx}}\times (\tfrac{OD=1}{3\times {10}^{7}cels/mL})|}_{Vrel=1}\times Vre{l}_{tx}\times (\tfrac{{10}^{6}\,\mu L}{1mL})$$

where Vrel_tx_ is the relative volume of cells at a certain time (tx) relative to the volume of cells at time 0.

In the same experiments, we collected samples and added cycloheximide to a final concentration of 100 μg/ml; measured absorbance; and imaged cells to quantify cell volume and reporter expression.

Intracellular glycerol determination was performed as previously reported^59^ with modifications. Cells were grown overnight in synthetic complete medium supplemented with NaCl 0.5 M at 26 °C. Cultures were then diluted in fresh medium and grown to mid-log phase until 1.0–1.5 × 10^7^ cells/mL. 10 mL of cell suspensions were placed at 37 °C in a water bath and 1 mL aliquots were withdrawn after 0, 10, 20 and 60 min. Immediately after taken the samples, cells were harvested by centrifugation (1 minute at 6000 g), resuspended in 1 mL of boiling water and incubated at 100 °C for 10 min. Samples were cooled down on ice for 10 minutes and subsequently centrifugated at 15000 g. Supernatants were used to measure glycerol and were stored at −20 °C. OD_600nm_ was determined at all time points. Glycerol concentration was determined using a commercial kit following manufacture’s indications (MAK117, Sigma-Aldrich). The amount of glycerol was normalized to OD_600nm_ and converted to intracellular glycerol concentration using a cellular volume of 40 fL and 2 × 10^7^ cell/OD. Data from 3 independent experiments were plotted versus time.

### Statistical methods

For statistical significance determination of fluorescence transcriptional reporters experiments (*P*_*STL1*_*-YFP*, *P*_*BMH2*_*-CFP* and *P*_*PRM1*_*-mCherry*), glycerol measurements and western blot data, we used one- or two-way ANOVA (using the aov() function of R) in experiments with at least three biological replicates. Where appropriate, we performed a Tukey’s HSD test (using the TukeyHSD() function of R). Error bar corresponds to the standard error of the mean (SEM). Statistical significance is indicated as follows: “ns” p > 0.1, “·” p < 0.1, “*” p < 0.05, “**” p < 0.01 and “***” p < 0.001. In all data with transcriptional reporters, number of yeast cells was greater than 200/data point.

## Electronic supplementary material

Supplementary Figures
