# Supplementary material for: Heat-stress triggers MAPK crosstalk to turn on the hyperosmotic response pathway
Source: Sci Rep. 2018 Oct 11;8:15168. doi: 10.1038/s41598-018-33203-6 (PMC6181916; doi:10.1038/s41598-018-33203-6)
Supplement: Supplementary file 1 — Supplementary Figures [file 41598_2018_33203_MOESM1_ESM.pdf]

Heat-stress triggers MAPK crosstalk to turn on the hyper-osmotic response pathway

Paula Dunayevich[1,2,4], Rodrigo Baltanás[1,2,4], José Clemente[1,2], Alicia Couto[3], Daiana Sapochnik[1,2,] Gustavo Vasen[1,2] and Alejandro Colman-Lerner[1,2]*

[1] Departamento de Fisiología, Biología Molecular y Celular, Facultad de Ciencias Exactas y Naturales (FCEN), Universidad de Buenos Aires (UBA), Buenos Aires, Argentina

[2] Instituto de Fisiología, Biología Molecular y Neurociencias (IFIBYNE), CONICET-UBA, Buenos Aires, Argentina

[3] CIHIDECAR-Departamento de Química Orgánica, FCEN, UBA, Buenos Aires, Argentina.

[4] Equally contributing authors

*Corresponding author. Tel: +54 11 4576 3368; E-mail: colman-lerner@fbmc.fcen.uba.ar

Figure S1

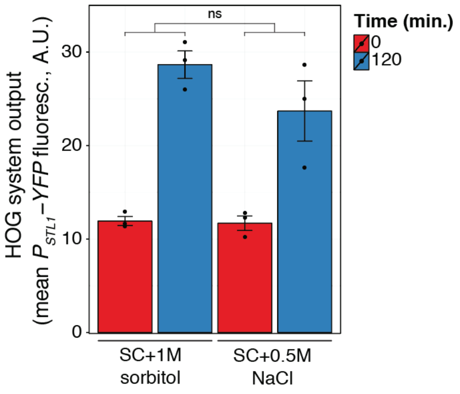

Supplementary Figure 1. High external osmolarity is required for HOG stimulation, not sorbitol per se.
HOG transcriptional reporter in WT yeast adapted to 1M sorbitol or 0.5M NaCl at 30°C and then transferred to 37°C for the indicated time. Values correspond to the mean of three independent replicates ± SEM. Two-way ANOVA showed no interaction between time and medium and did not find significant differences between 1M sorbitol and 0.5M NaCl.
Strain: LD3342.

Figure S2

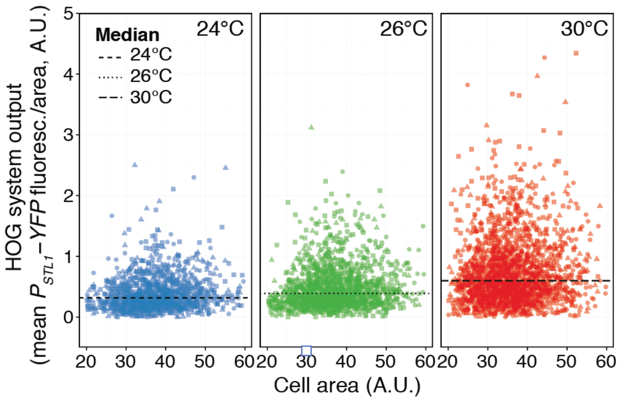

Supplementary Figure 2. Basal reporter expression correlates with growth temperature.
Scatter plot showing HOG transcriptional reporter in WT yeast adapted to 1M sorbitol at 24°C, 26°C or 30°C. Plot shows HOG system output/area vs. area of individual cells. Replicates are represented with different shapes.
Strain: LD3342.

Figure S3

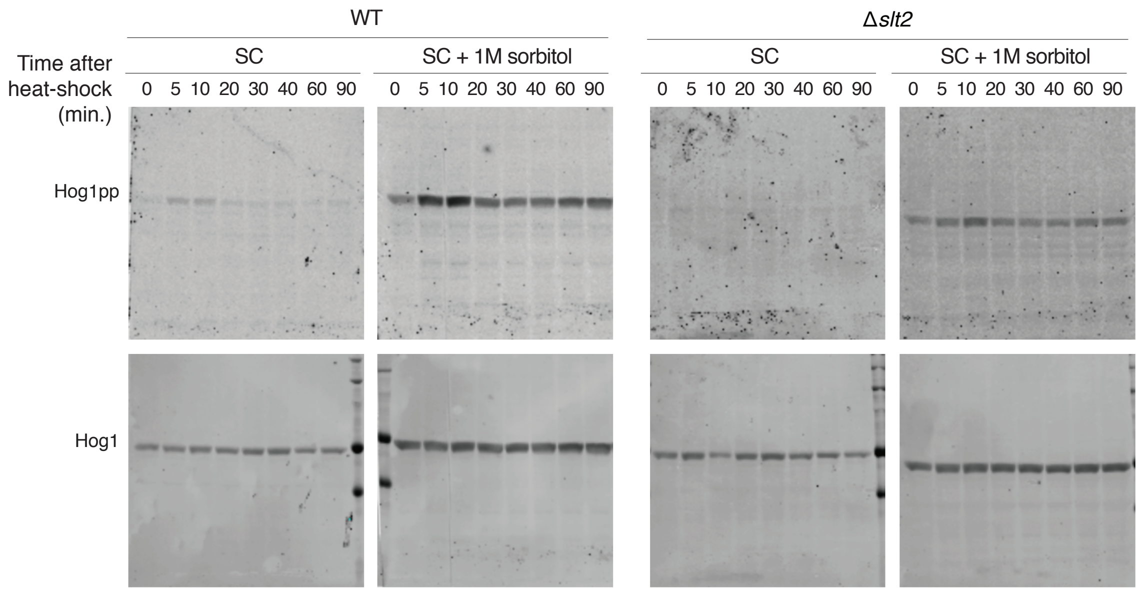

Supplementary Figure 3.
Uncropped western blots shown in Fig. 1F (Left) and Fig. 3A (Left and right).
Strains: LD3342 (WT) and RB3376a (Δ*slt2*).

Figure S4

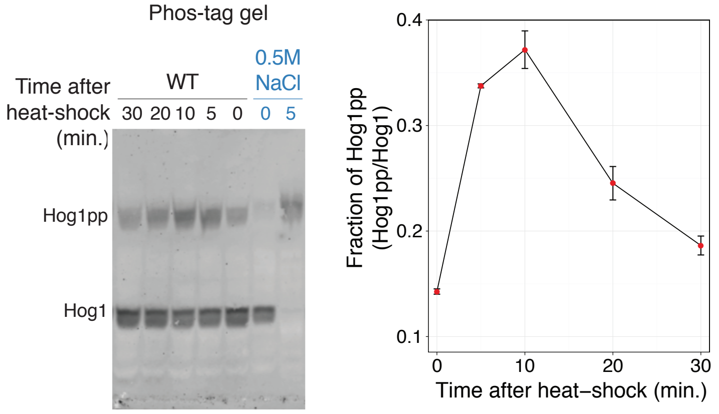

Supplementary Figure 4.
Uncropped phos-tag blot shown in Fig. 1G (Left) and fraction of phosphorylated Hog1 (Right). Values correspond to the mean of three independent replicates ± SEM.
Strain: LD3342 (WT).

Figure S5

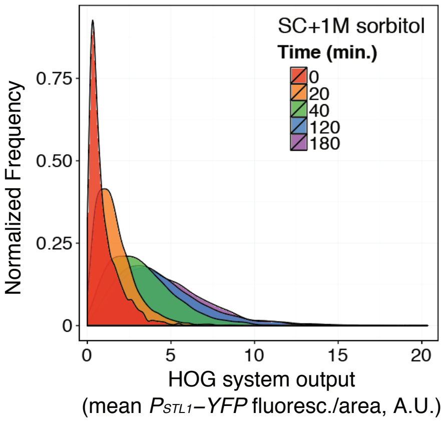

Supplementary Figure 5. HOG activation by heat-shock involves the whole population.
Histograms of the 1M sorbitol data as in Fig. 1C.
Strain: LD3342.

Figure S6

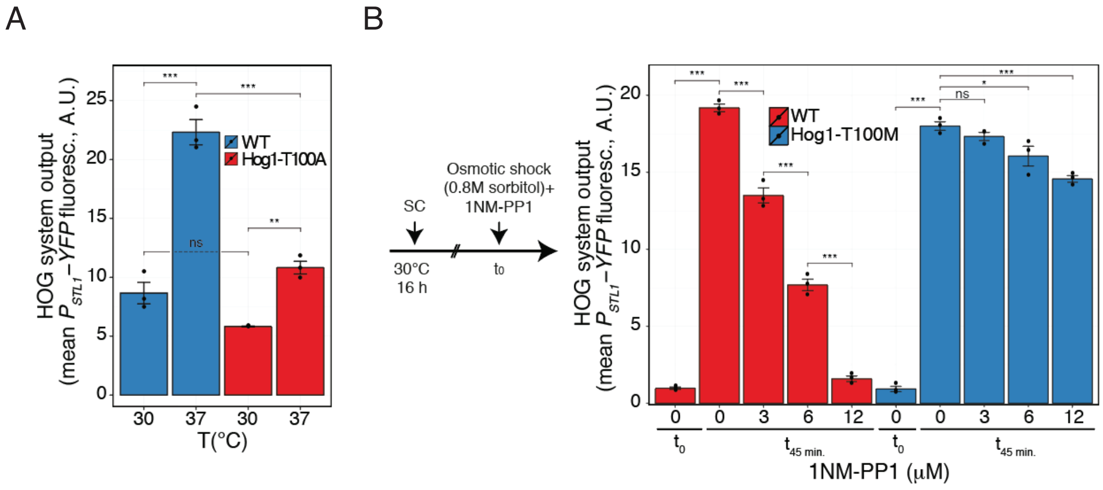

Supplementary Figure 6. HOG1-T100A strain is hypomorph. WT Hog1 is naturally sensitive to the inhibitor 1NM-PP1. HOG1-T100M is only slightly sensitive to 1NM-PP1.

(A) HOG transcriptional reporter in WT or HOG1-T100A mutant measured 2 hours after shift from 30°C to 37°C. Values correspond to the mean of three independent replicates ± SEM.

Strains: LD3342 (WT) and RB3937 (HOG1-T100A).

(B) HOG transcriptional reporter in WT or HOG1-T100M mutant grown in SC and shifted to SC supplemented with 0.8M sorbitol with increasing concentrations of 1NM-PP1. Left. Increasing concentrations of 1NM-PP1 progressively reduce high osmolarity stimulation of reporter protein. Right. The Hog1-T100M mutant is slightly sensitive to 1NM-PP1. HOG system output was measured after 45 minutes of hyperosmotic shock and values correspond to the mean of three independent replicates ± SEM.

Strains: LD3342 (WT) and RB3938 (Hog1-T100M).

Figure S7

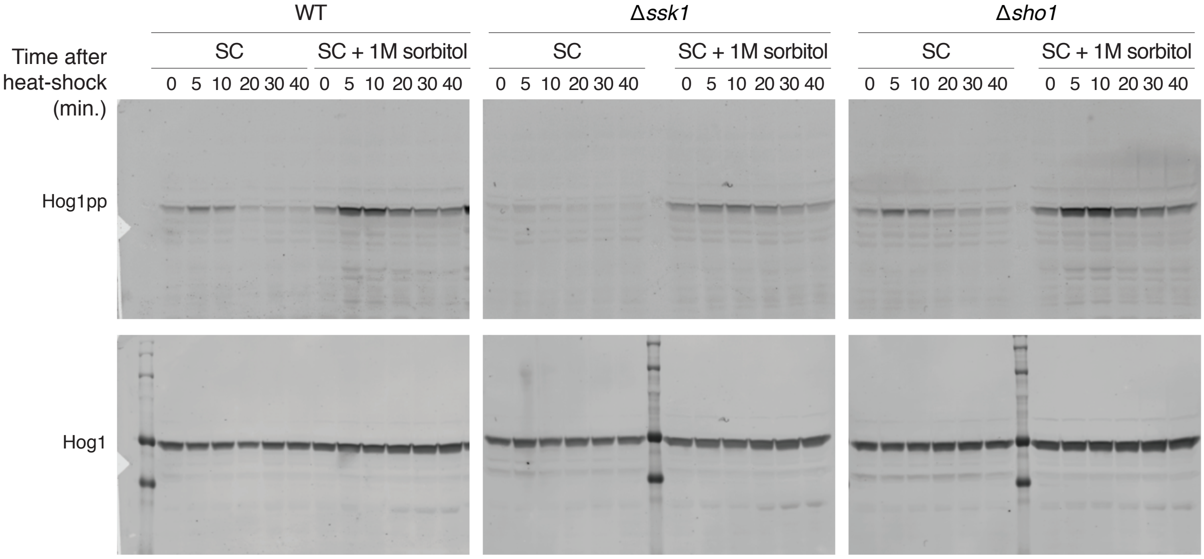

Supplementary Figure 7.
Uncropped western blots shown in Fig. 2A.
Strain: LD3342 (WT), RB3382 (Δ*ssk1*) and RB3704 (Δ*sho1*).

Figure S8

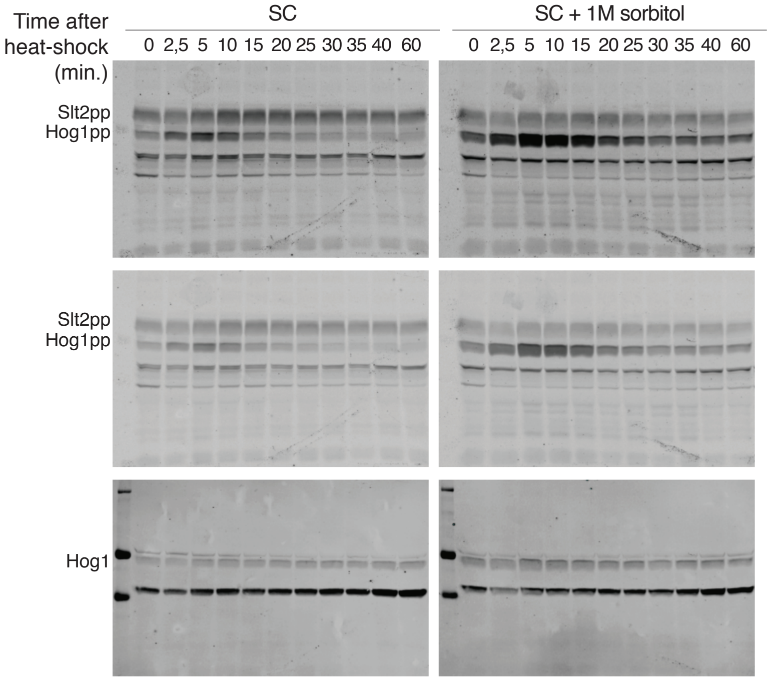

Supplementary Figure 8.
Uncropped western blots shown in Fig. 3D.
Strain: LD3342.

Figure S9

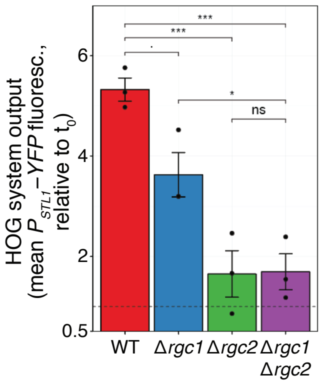

Supplementary Figure 9. Induction of the HOG reporter was greatly reduced in Δrgc2 and in the double knockout Δrgc1Δrgc2.

HOG transcriptional reporter in Fps1 channel regulator mutants. Values correspond to the mean of three independent replicates ± SEM, relative to t_0.

Strains: LD3342 (WT), RB3710 (Δ*rgc1*), RB3717 (Δ*rgc2*) and RB3722 (Δ*rgc1*Δ *rgc2*).

Figure S10

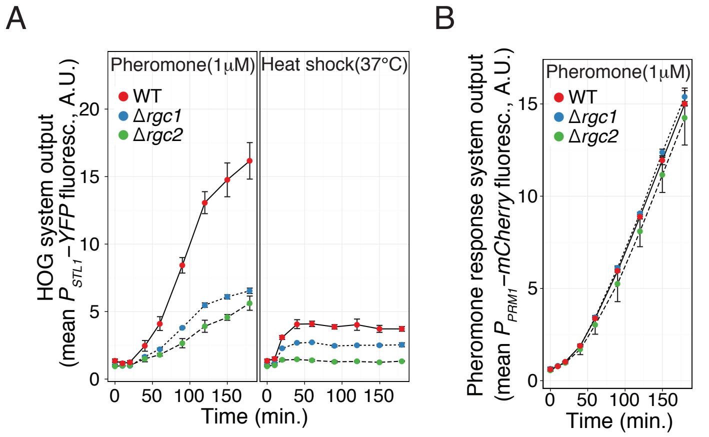

Supplementary Figure 10. Mating pheromone and heat-shock have distinct requirements for the Fps1 channel regulators.

(A) HOG transcriptional reporter in Fps1 channel regulator single mutants grown in SC+1M sorbitol and stimulated with 1 µM pheromone or shifted from 30°C to 37°C. Values correspond to the mean of three independent replicates ± SEM.

Strains: LD3342 (WT), RB3710 (Δ*rgc1*) and RB3717 (Δ*rgc2*).

(B) Pheromone pathway reporter (*PPRM1-mCherry*) in Fps1 channel regulator single mutants stimulated with pheromone as in A. Values correspond to the mean of three independent replicates ± SEM.

Strains: LD3342 (WT), RB3710 (Δ*rgc1*) and RB3717 (Δ*rgc2*).

Figure S11

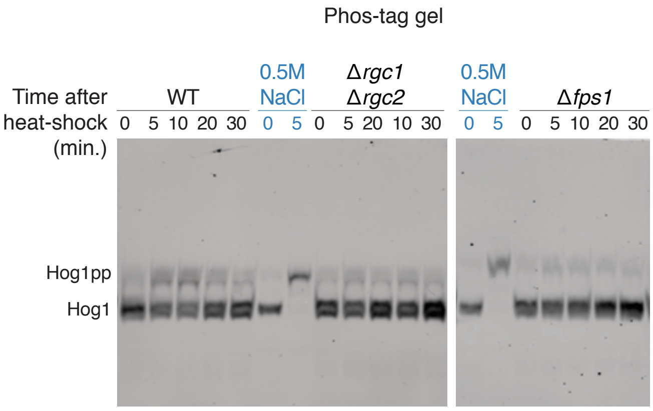

Supplementary Figure 11.
Uncropped phos-tag blot shown in Fig. 4B.
Strains: LD3342 (WT), RB3722 (Δ*rgc1*Δ*rgc2*) and RB3396 (Δ*fps1*).

Figure S12

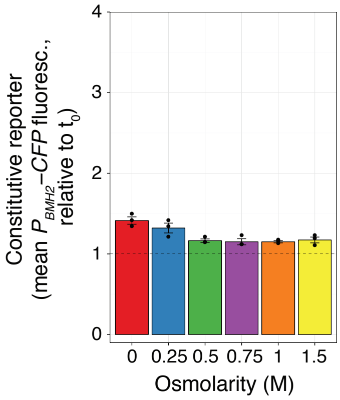

Supplementary Figure 12. Constitutive reporter expression was not affected by osmolarity.

Constitutive reporter expression (*PBMH2-CFP*) of the experiment shown in Fig. 4E (inset). Values correspond to the mean of three independent replicates ± SEM, relative to t_0.

Strain: LD3342.

Figure S13

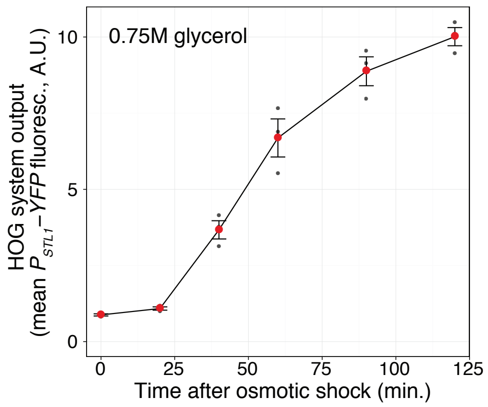

Supplementary Figure 13. Glycerol acts as an osmotic stressor when applied acutely.
Yeast grown in SC was shifted to SC supplemented with 0.75M glycerol. HOG system output (population average of the total YFP accumulated in each cell) vs. time correspond to the mean of three independent replicates ± SEM.
Strain: LD3342.

Figure S14

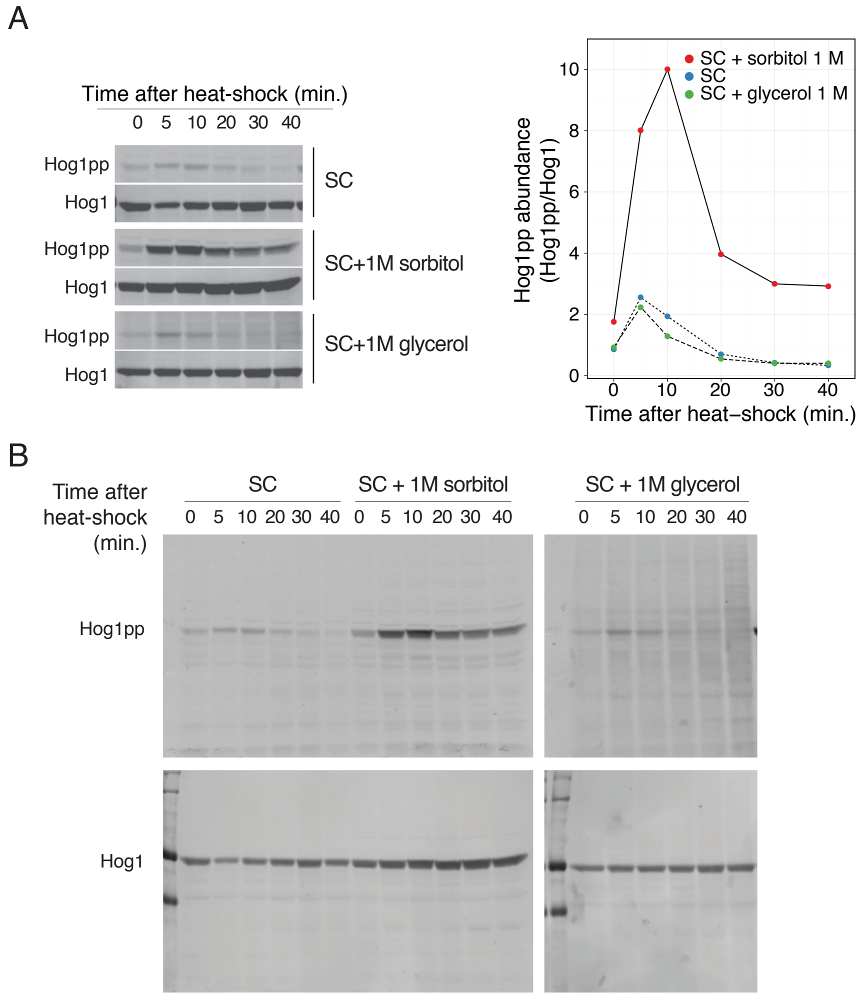

Supplementary Figure 14.
(A) HOG MAPK activation dynamics in WT grown in SC or adapted to SC+1M sorbitol or 1M glycerol following a shift from 30°C to 37°C. Left. Representative blot. Right. Quantification of phosphorylated Hog1.
Strain: LD3342.
(B) Uncropped blots shown in A.
Strain: LD3342.

Figure S15

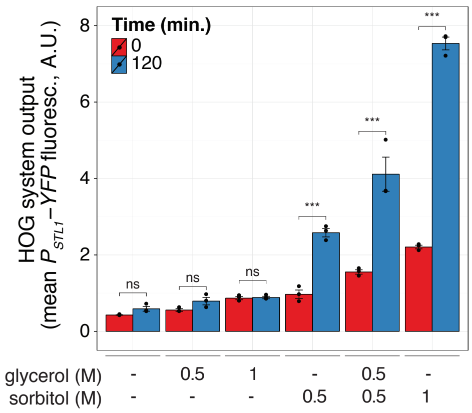

Supplementary Figure 15. Glycerol gradient regulates HOG activation.
Non-normalized data shown in Fig. 4G. Values correspond to the mean of three independent replicates ± SEM.
Strain: LD3342.

Figure S16

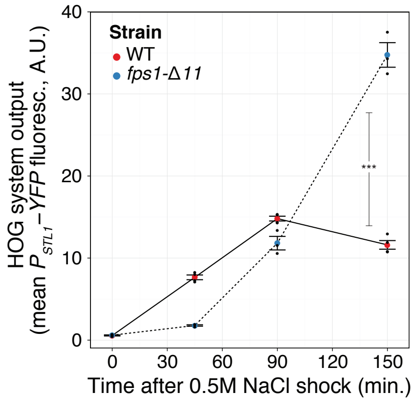

Supplementary Figure 16. *FPS1-Δ11* shows no apparent adaptation to a 0.5M NaCl shock.

Yeast grown in SC was shifted to SC supplemented with 0.5M NaCl. HOG system output (population average of the total YFP accumulated in each cell) vs. time correspond to the mean of three or more independent replicates ± SEM.

Strain: YPD6022 (WT) and YPD6023 (*FPS1-Δ11*).
